# Proteomics as a Complementary Technique to Characterize Bladder Cancer

**DOI:** 10.3390/cancers13215537

**Published:** 2021-11-04

**Authors:** Rubén López-Cortés, Sergio Vázquez-Estévez, Javier Álvarez Fernández, Cristina Núñez

**Affiliations:** 1 Research Unit, Hospital Universitario Lucus Augusti (HULA), Servizo Galego de Saúde (SERGAS), 27002 Lugo, Spain; Ruben.Lopez.Cortes@sergas.es; 2 Oncology Division, Hospital Universitario Lucus Augusti (HULA), Servizo Galego de Saúde (SERGAS), 27002 Lugo, Spain; Sergio.Vazquez.Estevez@sergas.es (S.V.-E.); Javier.Alvarez.Fernandez@sergas.es (J.Á.F.)

**Keywords:** proteomics, precision medicine, protein biomarkers, molecular classification, histoproteomics, histology, drug discovery, chemotherapy, urothelial cancer, bladder cancer

## Abstract

**Simple Summary:**

Although immunohistochemistry is a routine technique in clinics, and genomics has been rapidly incorporated, proteomics is a step behind. This general situation is also the norm in bladder cancer research. This review shows the contributions of proteomics to the molecular classification of bladder cancer, and to the study of histopathology due to tissue insults caused by tumors. Furthermore, the importance of proteomics for understanding the cellular and molecular changes as a consequence of the therapy of bladder cancer cannot be neglected.

**Abstract:**

Bladder cancer (BC) is the most common tumor of the urinary tract and is conventionally classified as either non-muscle invasive or muscle invasive. In addition, histological variants exist, as organized by the WHO-2016 classification. However, innovations in next-generation sequencing have led to molecular classifications of BC. These innovations have also allowed for the tracing of major tumorigenic pathways and, therefore, are positioned as strong supporters of precision medicine. In parallel, immunohistochemistry is still the clinical reference to discriminate histological layers and to stage BC. Key contributions have been made to enlarge the panel of protein immunomarkers. Moreover, the analysis of proteins in liquid biopsy has also provided potential markers. Notwithstanding, their clinical adoption is still low, with very few approved tests. In this context, mass spectrometry-based proteomics has remained a step behind; hence, we aimed to develop them in the community. Herein, the authors introduce the epidemiology and the conventional classifications to review the molecular classification of BC, highlighting the contributions of proteomics. Then, the advances in mass spectrometry techniques focusing on maintaining the integrity of the biological structures are presented, a milestone for the emergence of histoproteomics. Within this field, the review then discusses selected proteins for the comprehension of the pathophysiological mechanisms of BC. Finally, because there is still insufficient knowledge, this review considers proteomics as an important source for the development of BC therapies.

## 1. Introduction

In 2018, bladder cancer (BC) was the 12th most frequently diagnosed cancer [1]. In terms of age-standardized rate incidence per year, it accounted for 5–7% of all new male cases, and 2–2.5% of new female cases. The five-year prevalence was three to eight times higher than the incidence. Therefore, BC is one of the most prevalent cancers, turning it into a major burden for health care systems, which must deal with periodic medical check-ups and recurrent treatments.

Although BC is the most frequent neoplasia of the urinary tract (90–95%), it is not unique. Upper tract urothelial cancers (UTUCs, 5–10%), located between the renal pelvis and the ureters, and urethral cancer (<1%) are rarer forms [2]. Moreover, although about 90% of cancers that occur in the urinary tract are carcinomas of the transitional cells (hence, urothelial type tumors), less often, forms of non-urothelial types exist. These rarer forms account for up to 10–25% of the BC variants [3]. Moreover, a tumor injury of the bladder can be designated as papillary or flat according to the macroscopic structure. The former grows out towards the bladder lumen in branched finger-shaped protractions, whereas the latter spreads as a flattened mantle. Furthermore, both forms can be invasive or non-invasive, which is related to the tumor grade. In general, low-grade tumors rarely spread from their primary site, whereas high-grade tumors are more aggressive and assumed as invasive, requiring a more hard-hitting treatment strategy.

**Figure 1:**
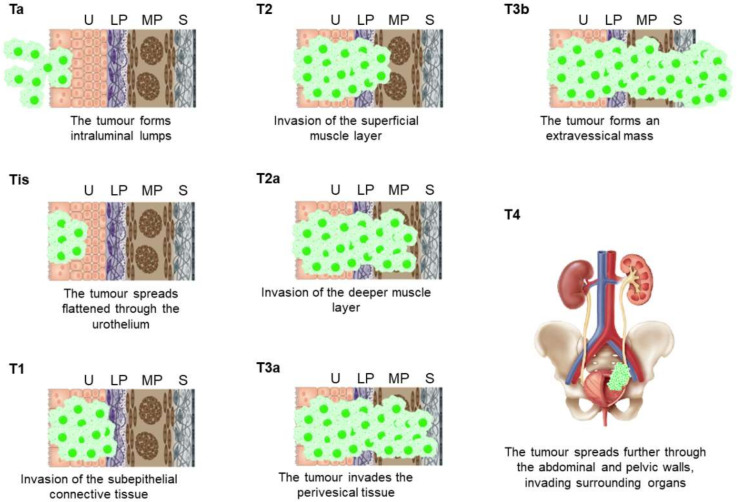
Schematic representation of the staging steps of bladder cancer according to the 2016 WHO (World Health Organization) TNM (tumor–node–metastasis) system. In the Ta stage, cancerous cells spread towards the lumen of the bladder in a papillary form, whereas in the Tis stage they form a flat mass embedded into the urothelium. In the T1 stage, the mass of cancerous cells invades the inner lining of connective tissue from the lamina propria. T2a and T2b stages refer to cancer invasion of the muscular layer of the bladder. In the T3a stage, the cancerous mass affects the serosa/adventitia, whereas in the T3b stage an extravesical, macroscopical cancerous mass surpasses the serosa/adventitia. In advanced T4 stages, the tumor spreads into surrounding organs of the genitourinary system and it can also migrate to the pelvic and abdominal walls. U: urothelium; LP: *lamina propria*; MP: *muscularis propria*; S: serosa/adventitia.

Urothelial BCs can be further classified according to their invasiveness as either non-muscle-invasive bladder cancer (NMIBC) or muscle-invasive bladder cancer (MIBC), a categorization closely connected to the TNM (tumor–nodes–metastasis) classification (Table 1). Tumors of the mucosa (Ta, and Tis), and tumors that have invaded the *lamina propria* (T1) are NMIBC. In contrast, tumors that have reached the bladder wall muscle (T2), the perivesical fat (T3), or nearby organs (T4) are grouped as MIBC (Figure 1). Although 75% of new BC cases are initially staged as NMIBC, 20–25% of these patients will recur, and another 10–20% of them will progress to MIBC within the first 5 years [4,5]. Moreover, although patients with primary or secondary MIBC are usually subjected to a similar first-line treatments, increased findings contradict this strategy because they usually have quite different responses and outcomes [6,7,8].

Histological staging of urothelial cancers is quite challenging, not only due to a high observer variability, but because two non-invasive types of lesions exist: papillary tumors (Ta), and carcinoma in situ (Tis) [9]. Moreover, discriminating a Ta lesion from a T1 NMIBC is challenging for the pathologist: between 25–34% of the tumor biopsies initially staged as T1 are later demoted to a Ta stage. Moreover, because about 10% of T1 cases will progress within the next 2 years, but fewer than 5% of Ta tumors do within a longer period of 6 years, correct identification is pivotal for managing valid clinical decisions [10]. These divergences can be related to the specific histology of the lining epithelium, hereby the importance of setting out a panel of protein biomarkers adapted to the histologist.

In addition, the 4th classification of genitourinary tumors was published by the WHO (World Health Organization) in 2016 [11,12]. Although it has great similarities with the previous version, some actualizations have been introduced regarding invasive urothelial tumor types (Table 2) [13,14]. Moreover, it is clearer that these invasive urothelial tumors diverge during their differentiation as frequently as in 33% of the cases, also being also more aggressive because they are associated with advanced stages and metastasis [15,16,17,18,19]. Again, researchers assert greater investigations to clearly identify these histological variants of BC for designing better and more personalized therapeutic schemes.

However, to date, cystoscopy remains the “gold standard”, and only a few biomarkers have been accepted for clinical practice in the last 20 years by the FDA [20,21]. Briefly, they are NMP22 (nuclear matrix protein 22), detected by ELISA; immunoCyt (uCyt+), which consists of a panel of fluorescent reporters to detect certain glycoproteins that are expressed solely on cancerous cells; UroVysion, a fluorescent in situ hybridization (FISH) assay that uses DNA probes to detect alterations on chromosomes 3, 7, and 17 or loss of the 9p21 locus; and BTA-TRAK, BTA-STA, which use monoclonal antibodies to detect the bladder tumor antigens (BTAs) complement factor H-related protein and complement factor H. A more systematized association between the histological variants with a specific panel of protein biomarkers would facilitate the classification of patients diagnosed with BC. Moreover, these protein panels would allow a better understanding of the molecular processes affecting cell plasticity, enable the cell potential to move and migrate from the primary tumor niche to surrounding tissues, and the relapse to therapy.

### The Pathways of Bladder Tumorigenesis

**Figure 2:**
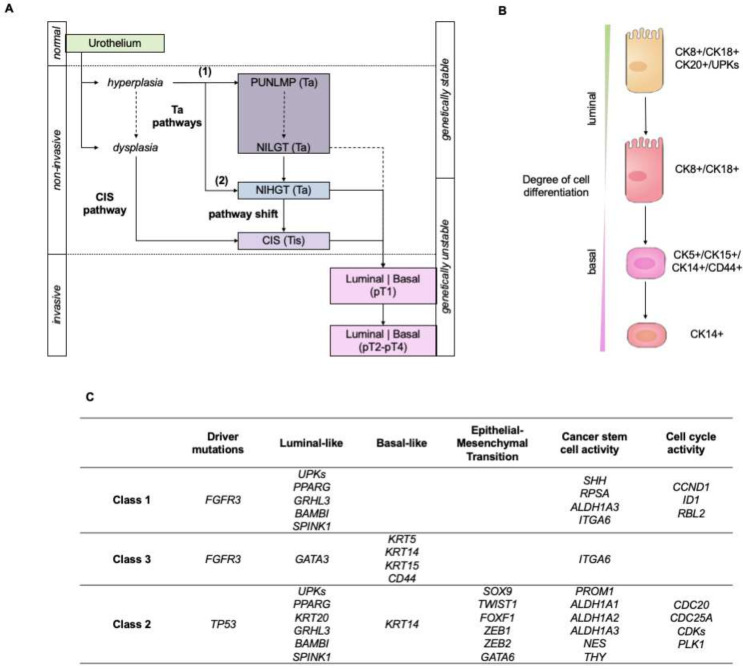
(**A**) Schematic representation of the potential pathways of bladder cancer tumor progression of bladder cancer pondering the and the ESMO (European Society for Medical Oncology) 2020 WHO (World Health Organization) 2016 staging classifications. PUNLMP: papillary urothelial neoplasm of low malignant potential. NILGT: non-invasive, low-grade tumor. NIHGT: non-invasive, high-grade tumor. CIS: carcinoma in situ. (**B**) Schematic representation of common changes in the expression of membrane-bound cytokeratins and uroplakins during the dedifferentiation of the urothelium in the early stages. It is a process where the epithelial cells lose their luminal differentiation to acquire a basal-like phenotype. (**C**) Summary of the representative molecular features for the Ta and the CIS pathways, including the class shift between them [22].

The initial stages of BC differ greatly at their genetic profile and clinical course levels; thus, Hedegaard, J. et al. proposed a three-way pathway to explain the progression from the Ta, Tis, and T1 NMIBC stages to MIBC [22]. Briefly, it can occur from either the Ta or the CIS (carcinoma in situ) pathway, the former being split into two subclasses, because the progression of one of the branches is firstly shifted towards CIS (Figure 2A).

Key differences in the genetic features have been observed between these three classes; class 1 and 3 showed mutations in *FGFR3*, characteristic of well-differentiated, low-grade urothelial carcinoma cells [23,24], whereas class 2 harbored mutations in *TP53*, characteristic of advanced, high-grade urothelial carcinoma cells [24,25]. Furthermore, classes 1 and 2 also share a high expression level of uroplakins—proteins which are also found in the differentiated luminal/umbrella cells. In contrast, class 2 tumors showed a high expression of KRT20—a protein biomarker linked to carcinoma in situ and to differentiated luminal/umbrella cells [26,27]. In class 3 tumors, it was found that KRT5 and KRT15 were overexpressed, also being biomarkers for intermediate-differentiated, basal cells [26,28]. However, the authors did not find a clear link to basal origin.

In contrast, tumors classified as class 2 and class 3 shared a high expression of KRT4—a cytokeratin that precedes the expression of KRT5 and that was described as a marker for epithelial cells in an undifferentiated state (Figure 2B) [26]. Class 2 was also enriched for biomarkers related to the epithelial–mesenchymal transition (EMT), and all three classes expressed biomarkers of cancer stem cell (CSC) activity (Figure 2C). Taken together, class 3 tumors did not only represent a shift from the Ta to the CIS pathway due to shared features with classes 1 and 2, but also a subset of cells with low cell-cycle and metabolic activity that has been associated with a dormant tumor state [22].

Other authors have also found this differentiation between either a luminal or a basal origin of BC [29]. The former is identified by alterations involving *FGFR3* and *KDM6A* mutations, whereas the basal origin is enriched in mutations affecting *RB1* and *NFE2L2*. In addition, BC usually shows affectation of the FGFR3/RAS or the TP53/RB1 signaling pathways, with mutations in *FGFR3* frequently occurring during the hyperplasia of the urothelium, and mutations in *TP53* facilitating the transition from dysplasia to invasion via the CIS pathway [30]. Mutations in *RB1* may appear later, thus, they would allow the progression from urothelial hyperplasia to CIS [31], which supports the shift during tumorigenesis already explained above.

Another study further investigated the existence of two subtypes in Ta tumors in NMIBC by genome-wide analysis, copy-number profile, mutation burden and gene expression profile [32]. The authors proposed two subgroups: GS1, with no or few copy-number variations; and GS2, with a deletion of chromosome 9q, an affectation of the mTORC1 signaling pathway, an altered glycolysis rate, and mutations in the tumor suppressor gene *KDM6A* [33]. This mutation was more frequently found in women (74%) than in men (42%), and the authors used it to investigate the biased incidence that BC displayed according to the gender (male/female ratio of 3:1) [33].

**Figure 3:**
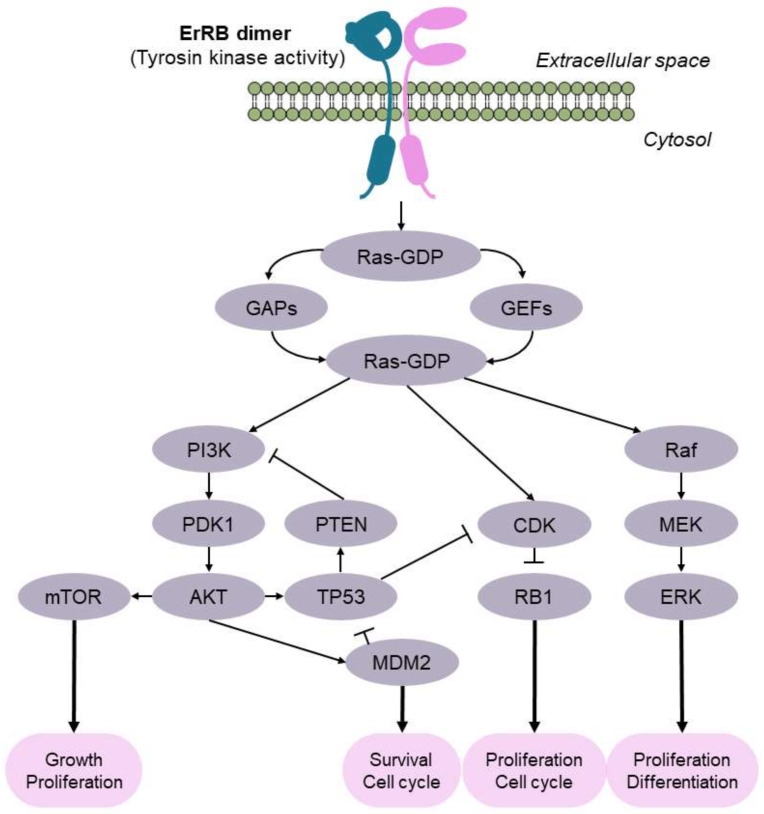
Schematic representation of the signaling pathways more commonly affected in bladder cancer. RAS signaling is associated with the ErRB dimer receptor, which is also a tyrosine kinase receptor frequently mutated in bladder cancer.

Common genetic variations in BC are mutations in *FGFR3*, *TP53,* and *RB1* genes [34]. Up to 80% of NMIBC and 10–20% of MIBC cases report activating mutations in *FGFR3*, which have been associated with a higher risk of recurrence because FGFR3 is a receptor that activates cell proliferation via the RAS/MAPK pathway (Figure 3). In addition, about 10% of BC cases also harbor mutations in RAS genes [35,36]. The TP53/RB1 pathway is key to regulating the progression of the cell cycle (Figure 3). Most (89%) MIBC patients exhibit inactivated TP53/RB1 signaling, in which almost 50% of them have a direct affectation of the *TP53* gene. *RB1* mutations are in second place, with 17% of MIBC cases [34]. In addition, 29% of MIBC cases harbor mutations in *CDKN2A*, a negative regulator of the RB1 pathway. Interestingly, the affectation of mTORC1 also involves dysregulation of the PIK3/AKT/mTOR signaling pathway, which communicates with the ErRB family of receptors, and solapates with the mediation of RAS (Figure 3). The PIK3/AKT/mTOR pathway participates in the cell cycle and is directly related to cell proliferation and longevity. ErRB activation promotes this signaling pathway. Mutations in *ERBB2*, *ERBB3,* and *PIK3CA* are common (12%, 10%, and 22% of MIBC, respectively) [34]. Moreover, mutations inhibiting the function of *PTEN*, which acts as a negative regulator, are also common [37]; in fact, a reduced expression of PTEN—measured by immunohistochemistry—was associated with those BC patients who showed a higher progression and recurrence of their disease [38].

## 2. Omics Sciences in the Molecular Classification of Bladder Cancer

Although cancer is considered an essentially gene-based disease, it is impossible to predict the exact activity or concentration of proteins by simply approximating the mRNA levels, because the expression and activity of proteins may be modulated through mRNA maturation processes or by PTMs (post-translational modifications). All these modifications in the protein content or structure profoundly affect its function and lead to protein isoforms that may vary between different stages, and even from niche-to-niche or cell-to-cell. Hence, the goal of classifying bladder cancer into subtypes can be performed from the genomic or the proteomic perspective, both with some inherent limitations. Moreover, the former may consider the existence of not only mRNA as part of the transcriptome, but also long non-coding (lncRNA) or interfering (miRNA) sequences.

### 2.1. Molecular Classifications of Bladder Cancer Based on the Transcriptome

**Figure 4:**
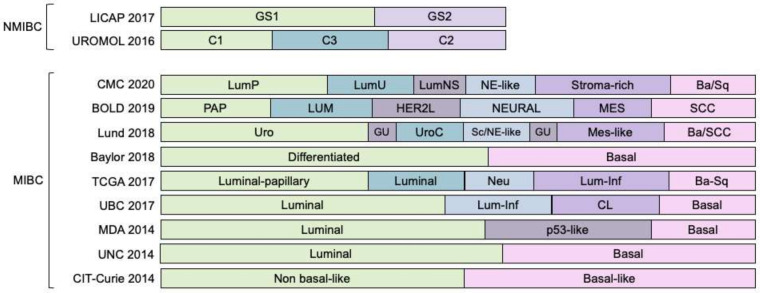
Schematic representation of the similarities between different molecular classifications of bladder cancer. For NMIBC, molecular subtypes were proposed by the Leeds Institute of Cancer and Pathology (LICAP 2017), and the European UROMOL project (UROMOL 2016). GS1 = genomic subtype 1, GS2 = genomic subtype 2, and C1 = class 1, C2 = class 2, C3 = class 3. For MIBC, nine molecular classifications have been suggested to date, with a consensus molecular classification presented in 2020 (CMC 2020): LumP = luminal papillary, LumU = luminal unstable, LumNS = luminal non specified, NE-like = neuroendocrine-like, Ba/Sq = basal/squamous. Cancer Science Institute of Singapore (BOLD 2019): LUM = luminal-like, PAP = papillary-like, HER2L = HER2-like, NEURAL = neural-like, MES = mesenchymal-like, SCC = squamous-cell carcinoma-like. The Lund University (Lund 2018): Uro = urothelial-like, GU = genomically unstable, UroC = urothelial-like C, Sc/NE-like = Small-cell/neuroendocrine-like, Mes-like = mesenchymal-like, Ba/SCC = basal/squamous-cell carcinoma. The Baylor College of Medicine (Baylor 2018): Differentiated, and Basal. The Cancer Genome Atlas Network (TCGA 2017): Luminal-papillary, Luminal, Neu: neuronal, Lum-Inf: luminal-infiltrated, Ba-Sq: basal-squamous. The University of British Columbia (UBC 2017): Luminal, Lum-Inf = luminal-infiltrated, CL = claudin-low, and Basal. The MD Anderson Cancer Center (MDA 2014): Luminal, p53-like, and Basal. The University of North Carolina (UNC): Luminal, and Basal. The Institut Curie (CIT-Curie 2014): Non-basal-like, and basal-like [39].

It is expected that the molecular characterization of BC will reveal important information for stratification patients not only in terms of an early-stage cancer progression but also for predicting clinical outcomes and designing the most effective treatment, because not all tumors will respond in the same manner despite falling in the same classical clinical or anatomopathological staging and grading descriptors that were already depicted; thus, great efforts have been made and to date, nine molecular classification systems of MIBC and two of NMIBC have been proposed (Figure 4) [39].

Sjödahl, G. et al. presented a six-tiered classification, based firstly on clustering the coding transcriptome from 308 samples, and further reordered into five subtypes and eight subgroups plus one outlier after considering the tumor cell phenotype by immunohistochemistry [40,41,42,43]. It is known as the Lund taxonomy, with five subtypes: Uro (urothelial-like), GU (genomically unstable), Basal/SCC (basal/squamous carcinoma cells), Mes-like (mesenchymal-like) and Sc/NE-like (small-cell/neuroendocrine-like). The Uro subtype is further subdivided into: UroA, UroA-Prog(ressed), Uro-Inf(iltrated), UroB, and UroC. The GU, and Basal/SCC-like subtypes have two subgroups each to consider the absence (GU, and Ba/Sq) or the existence of infiltration (GU-Inf, and Ba/Sq-Inf). Mes-like and Sc/NE-like do not have any subgroup, and an outlier group is merely named as infiltrated (Inf) because cell infiltration was so great to unequivocally identify any subtype. UroA, and UroA-Prog (UroA-progressed) correspond to NMIBC patients, with the UroA-Prog subgroup being a cohort of cystectomized patients. Apart from this feature, the genomic profile was coincident, with high expression of the transcripts *FGFR3*, *CCND1*, *RB1*, *PPARG*, *GATA3*, and *TP63*, and low expression of the transcripts *CDKN2A,* and *ERBB2*. The major difference is at the level of cell proliferation—UroA-Prog is more aggressive than UroA. The UroB subgroup is similar to UroA (high expression of *FGFR3*, *CCND1*, and *RB1*, and low *CDKN2A* expression), with the difference that UroB shows a stratified cell organization with basal/squamous differentiation, and the expression of basal markers such as *KRT5*, *CDH3*, and *EGFR*. Despite this basal profile, UroB is not considered the progenitor of the Ba/Sq subtype, but a subgroup that acquired these molecular features in a manner that reflects the findings made by Hedegaard J et al. in the early stages of NMIBC [22]. UroC also represents a further progressed variant of UroA with a similar expression of *FGFR3*, although it also resembles GU tumor subtype in terms of *ERBB2* expression. Thus, the difference between UroB and UroC subgroups is that the former attains Basal/SCC-like (basal/squamous) features and the latter shows a progression to the GU gene expression profile [41]. In contrast to the higher heterogeneity within the urothelial-like subtype, GU and Basal/SCC-like subtypes are simply subclassified considering the presence of infiltrated cells that underwent epithelial–mesenchymal transition [41].

The classification of CIT-Curie is based on a 40-gene expression classifier of 85 MIBC patients. Two subgroups were identified, highlighting the basal-like cluster, identified by the overexpression of markers such as *KRT5*, *KRT6A*, *KRT14,* and *EGFR*, which are associated with cell undifferentiation and a potential response to EGFR-targeted immunotherapy, and a low expression of uroplakins (*UPK1A*, *UPK1B*, *UPK2,* and *UPK3A*), which are common biomarkers of a differentiated urothelium [44,45].

The MD Anderson Cancer Center (MDA) presented a three-tiered system from a cohort of 73 patients, later enlarged to five: luminal, luminal-p53, basal, basal-p53 and the double negative class [29,46,47]. As in other cited works, luminal tumors were characterized by an expression signature similar to the intermediate/superficial layers of a normal urothelium, with upregulation of *FGFR3*, *ERBB2*/3, *GATA3*, *PARPG*, *KRT18*, *KRT19*, *KRT20*, *KRT8*, and *KRT9*. Basal tumors exhibited an expression profile similar to the basal layer of the urothelium, with upregulation of *KRT14*, *KRT16*, *KRT5*, *KRT6A*/*6B*/*6C*, and the enrichment of mutations in *TP53* and *RB1*. The p53 subgroup is present in both the luminal and basal subtypes, and strongly correlates with UroB, GU, and infiltrated subgroups of the Lund taxonomy [46]. In addition, it was characterized by mutations in *TP53* and changes in the mRNA expression, high cell infiltration, aggressive behavior, and tended to be chemoresistant [46]. The double-negative subtype comprises a small subset of cases (4%) that did not express either luminal or basal markers, but a low expression signature of claudin-related genes (*CLDN3*, *CLDN4*, *CLDN7*, *CDH1*) and high expression of *VIM*, *SNAI2*, *TWIST2*, and *ZEB1*/*2*.

The UNC (University of North Carolina) classification found that their subtypes reflected the hallmarks of breast cancer biology, especially regarding the low claudin expression, which was also investigated by other groups [48]. A two-tiered system was proposed after investigating a dataset of 262 patient cases and validating it with 49 tumors from the Memorial Sloan-Kettering Cancer Center [49]. Luminal and basal clusters differentiated themselves based on urothelial differentiation biomarkers consisting of uroplakins (*UPK2*, *UPK1B* and *UPK3A*) and cytokeratins (*KRT5*, *KRT6B*, *KRT14* and *KRT20*). The claudin-low was also identified as a subset of the basal subtype with increased frequency of *EGFR* amplification, low rates of mutations of *FGFR3* and *KDMA6,* and an active immunosuppressed profile that points to potential responses to immunotherapy [50].

Focusing on the urothelial differentiation pathway, the Baylor classification system developed an 18-gene expression signature assigning NMIBC and MIBC patients to either a basal subtype or a differentiated subtype [51]. The former expressed a higher level of cytokeratins related to the undifferentiated basal cells found in a healthy urothelium, and the latter exhibited the opposite behavior, with an underexpression of these basal biomarkers, pointing towards a differentiated status of the tumor cells. This reversion of the phenotype is usually related to a more aggressive cancer; therefore, the basal subtype also had the worst overall survival rate.

TCGA (The Cancer Genome Atlas) research network initially proposed a four-tiered classification, with groups referred to as I–IV, based on 131 patients, whole-transcriptome sequencing, targeted protein analysis of papillary/squamous features, urothelial differentiation, and *ESR2* and *ERBB2* gene status, and gene and protein expression [52]. This classification was further updated using 412 cases and introduced the analysis of copy-number variations, whole-genome sequencing, whole-exome sequencing, RNA-Seq, methylome and protein expression. Five clusters were proposed: luminal-papillary, luminal-infiltrated, luminal, basal/squamous, and neuronal [34].

Nevertheless, it has become evident that the task of proposing a consensus molecular classification is far more complex than initially; comparative analysis between different classification proposals showed different levels of overlapped clusters (Figure 4) [53]. Even so, efforts are being made in this direction, with three recent publications curating larger cohorts of patients and contrasting these previous proposals [54,55,56].

From a cohort of 343 patients from the classification systems of UNC, MDA TCGA and Lund, the UBC (University of British Columbia) classification system identified two main subtypes further divided into four subgroups of MIBC: the basal subtype, with a claudin-low subset; and the luminal subtype, with a luminal-infiltrated subset [54].

The BOLD (bladder carcinoma subtypes of large meta-cohort database) molecular classification compiled 2411 samples from NMIBC and MIBC patients: the former from UROMOL 2016 and LICAP, and the latter group from the UNC, MDA, TCGA 2017, UBC, and Lund proposals [55]. The authors produced a six-class system based on whole gene expression and gene mutational status, MC1/Neural (neural-like), MC2/Lum (luminal-like), MC3/Pap (luminal-papillary), MC4/HER2L (HER2L-like), MC5/SCC (squamous cell carcinoma) and MC6/Mes (mesenchymal), with close inter-relationship with previous proposals. To date, BOLD is the only molecular classification system taking into account NMIBC and MIBC patients, proving good reproducibility of these six subtypes in independent NMIBC, MIBC, and metastatic BC cohorts [55]. Interestingly, the authors also found an association between these six classes and the standard clinicopathological classification. The six clusters are well represented within the urothelial type, whereas most non-urothelial types were represented by the MC5/SCC, more specifically, sarcomatoid and squamous tumors. MC4/HER2L and MC6/Mes accounted for most of the micropapillary histological variants cases, glandular tumors displayed similarity with MC5/SCC and neuroendocrine tumors corresponded mainly with MC1/Neural.

In contrast, the CMC 2020 (Consensus Molecular Classification) compiled 1750 MIBC transcriptomic profiles to compare the classifiers from six previous classification systems (Baylor, UNC, MDA, TCGA, CIT-Curie and Lund), converging on six biologically relevant classes, which authors labelled as LumP (luminal-papillary), LumNS (luminal nonspecified), LumU (luminal unstable), Stroma-rich, Ba/Sq (basal-squamous) and NE-like (neuroendocrine-like) [56]. Again, the authors also found a strong correlation between these six classes and the classical histological patterns. LumP tumors closely resemble papillary variants, LumNS tumors to micropapillary variants, and, more specifically, to carcinoma in situ, whereas LumU and stroma-rich tumors were harder to correlate, although fall into micropapillary variants. Ba/Sq class included 79% of tumors with histological squamous differentiation, although it also extended to sarcomatoid variants. NE-like tumors showed neuroendocrine differentiation in 72% of the cases.

Due to the complexity of the tumor environment, and with frequent infiltration of stromal and immune cell populations, it is difficult to assume the singularity of a tumor subtype by relying solely on genomics, especially when dealing with MIBC as they have one of the highest mutation burdens, with strong immunogenicity [57,58]. By detecting 28 protein markers by immunohistochemistry, Sjödahl, G. et al. showed the existence of aberrant and inconsistent protein expression within a tumor subtype [41,42]. These deviations were a consequence of non-tumor cell infiltration, and the authors suggested they should be accounted to explain the existence of pseudo-differentiation within that molecular subtype. Consequently, proteomics has already been revealed as a promising technique to elucidate the broad complexity of the molecular mechanisms governing BC, with the great potential to propose alternative classifications too.

### 2.2. Molecular Classifications of Bladder Cancer Based on Proteomics

Interestingly, a proteomic analysis by RPPA using a list of 208 antibodies allowed the clustering of 343 primary tumors in five MIBC subtypes (i.e., epithelial/papillary, epithelial/intermediate, proliferative/low signaling, EMT/hormone signaling, and EMT reactive) [34]. They not only presented a different profile but, in addition, patient clusters had different outcomes in OS. A key difference with the molecular classification of TCGA 2017 by RNA-Seq is that these five clusters showed a frequency of ≈20%. Moreover, despite showing some similarities with the molecular classification by RNA-Seq, the proteomic clusters included several RNA-Seq clusters. In this regard, the epithelial/papillary cluster (22%) includes most of the luminal-papillary subtypes cases, and it showed the best patient outcome and low expression of EMT markers. The epithelial/intermediate cluster (22%) includes cases classified as luminal-papillary, luminal-infiltrated and luminal, and it is characterized by elevated HER2 expression, supporting a potential use of anti-HER2 therapies with these patients. The proliferative/low signaling cluster (20%) includes the basal-squamous subtype and the three luminal subtypes. This cluster has a high activation of the cell cycle pathways, with a high expression of CYCLINB1 (Cyclin-B1) and PCNA (proliferating cell nuclear antigen), with affectations in the PIK3/AKT/mTOR signaling pathway. These tumors also have a high expression of EGFR; thus, these patients may benefit from therapies with EGFR inhibitors. The EMT/hormone signaling cluster (15%) is quite heterogeneous, with all five subtypes represented within this cluster, and is the group with the worst outcome. Hormone signaling is activated via activation of the phosphorylation of the Src kinase, which regulates the hormone-dependent nuclear traffic and cell cycle progression [59]. The EMT cluster (22%) is also quite heterogeneous, with all five subtypes represented within this cluster. Despite its name, it did not show a worse outcome compared to the previous cluster. This subtype expresses proteins that may be related to interactions between cancers cells and their microenvironment, including other cells, such as fibroblasts. EMT/hormone signaling and EMT clusters show high expression levels of HSP70 (heat shock 70 kDa protein), fibronectin, collagen-VI, annexin-1, and caveolin-1. Dysregulation of fibronectin, collagen-VI, and caveolin-1 correlates with the progression of EMT because they participate in the extracellular matrix or cell cytoskeleton and, hence, in cell mobility [60,61,62].

A similar scheme was developed by de Velasco, G. et al. by evaluating the proteome of 58 MIBC patients using FFPE tumor tissue samples [63]. In that study, 4405 proteins were identified, of which 1453 were detected in at least 75% of the samples, with a subset of 34 proteins being differentially expressed in two clusters. Cluster 1 was defined by the overexpression of 20 proteins related to focal adhesion, the extracellular matrix, and the regulation of metabolics such as glycolysis and pyruvate metabolism. On the other hand, cluster 2 showed an overexpression of proteins mainly related to transcriptional processes and the immune response. Additionally, it also exhibited increased activity of cell processes such as RNA processing, transport of vesicles, and the proteasome.

More recently, the proteome of a group of 117 patients with a primary diagnosis of BC (98 with NMIBC, and 19 with MIBC) was studied with hyphenated liquid chromatography-tandem mass spectrometry (LC-MS/MS) [64]. As far as we know, this is the first time that the proteome of BC patients was investigated using a mass-based proteomics technique, thus enabling the mass identification of proteins. As will be discussed in the next section of this review, mass-based proteomics techniques cope with broader entities than antibody-based proteomics techniques without the necessity for previous knowledge of the proteins of interest. By clustering the proteome of NMIBC patients, the authors proposed the existence of three NMIBC proteomic subtypes (NPSs). NPS1 was the smallest cluster, showing features of a highly aggressive subtype, and being close to the proteome of MIBC patients. It was characterized by the expression of proteins related to the regulation of the immune system, inflammation, cell proliferation, the unfolded protein response, and DNA damage response. The NPS2 subtype was characterized by an enrichment of protein biomarkers related to cell infiltration, and EMT (epithelia–mesenchymal transition). NSP3 largely resembles low aggressive luminal cancers, with high levels of KRT20, CDH1, and UPKSs, and affectation of the glycolytic pathway. In brief, a total panel of 77 proteins could significantly discriminate between these three proteomic subtypes. More importantly, this study also supports consistency between the data at the proteome level and the molecular clustering using the information at the mRNA level.

Despite the variable number of clusters in the three aforementioned publications, some common assumptions may be made. Firstly, the number of samples is critical, because with about 50 samples, only 2 clusters were identified by Velasco, G. et al., whereas the TCGA identified 5 clusters using almost 350 patients. The low number of MIBC patients recruited by Stroggilos, R. et al. was insufficient to segment them. Secondly, the clustering method affected the number of clusters. However, the affected mechanisms and pathways remained highly persistent within the three publications. Specifically, these included: EMT and cell adhesion, regulation of the cell cycle, stromal microenvironment and the extracellular matrix, interactions with the immune system, cell metabolism, DNA repair, intracellular trafficking and cell stress, and receptor cell signaling.

## 3. Overview of the Proteomics Techniques, and Novel Advances in the Field

**Figure 5:**
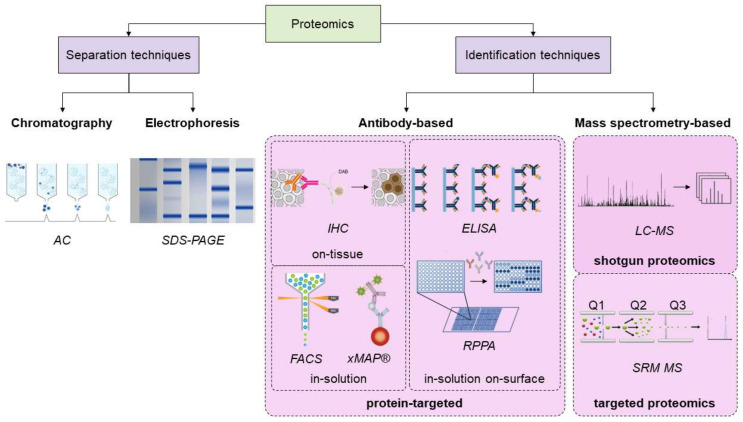
Schematic representation of proteomics techniques. Separation techniques are usually employed in the preparative steps for simplifying the complexity of the proteome. AC (affinity chromatography) is based on the differential adsorption of protein onto the surface and SDS-PAGE (sodium dodecyl sulfate-polyacrylamide gel electrophoresis) relies on the motion of proteins under the influence of an electric field. Antibody-based techniques identify proteins while maintaining the cell/tissue integrity, such as IHC (immunohistochemistry), FACS (fluorescence-activated cell sorting) flow cytometry or xMAP (multi-analyte profiling), or losing it, such as ELISA (enzyme-linked immunosorbent assay) or RPPA (reverse-phase protein assay). Mass spectrometry-based techniques cope with a higher number of proteins than antibody-based techniques and can be classified as shotgun proteomics when the objective is to identification, with the possibility of quantification them, such as LC-MS (liquid chromatography-mass spectrometry), or targeted proteomics, whether the purpose is to accurately quantify the expression levels of a set of proteins, such as SRM MS (single reaction monitoring mass spectrometry).

Protein analytical techniques can be divided into two main groups: those aimed at protein separation and those aimed at protein identification. Within the former group, we can cite well-known techniques, such as chromatography or electrophoresis. The latter group comprises those tools based on the use of antibodies or mass spectrometry (Figure 5). Antibody-based techniques can also be considered because protein-targeted techniques as they require prior knowledge about the protein(s) of interest to select the proper antibody. They are quite easy to perform and implement in a lab; thus several workflows have been developed. Briefly, they can be classified into three main groups. On-tissue antibody-based techniques such as IHC (immunohistochemistry) leverage the tissue slice as the basis for exposing the protein(s) of interest to the antibodies. These methods offer the additional advantage of maintaining histological integrity. In-solution on-surface methods also require a solid platform to allow the binding between an antibody and its antigen. ELISAs (enzyme-linked immunosorbent assay), and Western blots are well-known techniques. Finally, in-solution antibody-based techniques do not require any solid platform and the interaction of antibodies with their antigens occurs in solution. The main advantage of these methods such as FACS (fluorescence-activated cell sorting) flow cytometry is they are well adapted for the continuous flow of work, i.e., they can be easily automated by fluidics.

Although these antibody-based, protein-targeted techniques can quantify protein levels with high accuracy, they are highly laborious and costly. Moreover, they have not been conceived for multiplexed assays beyond few analytes, rarely reaching ten, although this concern was partially solved in recent years with the development of xMAP (multiple analyte profiling by multiplexed particle-based flow cytometric assay), RPPA (reverse-phase protein array), or mIHC (multiplexed immunohistochemistry) [65,66,67,68,69,70].

In contrast, proteomics-based mass spectrometry techniques are more straightforward because they allow the identification and quantitation of hundreds of proteins in just a single run in a manner that even multiplexed antibody-based techniques cannot approximate to date. They have progressed considerably over the last years and have replaced some of the aforementioned methods in the discovery of cancer biomarkers—although applications in clinical research are still one step behind. In addition, they have been traditionally classified as shotgun proteomics and targeted proteomics (Figure 5).

Shotgun proteomics, also called discovery-based proteomics, is the most widely used approach. It consists of obtaining a broad list of hundreds or even thousands of identified proteins, which can also be quantified in comparative analysis to address which of them might be down- or up-regulated. This quantitation can be performed using label-based technologies or label-free-based technologies, which take advantage of the precursor signal intensity or spectral counting. However, although shotgun proteomics is a powerful tool in the field of basic biomedical research, it has usually fallen short in providing useful translational and clinically relevant information because of the huge complexity of data and difficulties to propose and evaluate surrogate endpoints. In contrast, targeted proteomics allows for the quantitation of several proteins of interest in similar experimental conditions to shotgun proteomics, albeit performing relative or absolute quantification with high specificity and sensitivity, even in particularly complex backgrounds, tracing the specific pattern of the fragmentation of the parent ions. It requires identifying the proteotypic peptides (peptides with unique amino acid primary sequences that identify a specific protein); therefore demands prior knowledge of the proteins of interest. This is the reason why this strategy usually follows a hypothesis-driven procedure. The advent of targeted proteomics has also helped mitigate the problem of the background and to ameliorate the intrinsic stochastic effects that govern the parent peptide ionization that is difficult to reproduce in shotgun proteomics.

Regarding proteomics-based mass spectrometry, we need to consider that this approach deals with a protein abundance that, in human cells, spans from 1 to 10^7^ copies per cell, and about 20,000 different proteins expressed at a given time, whereas the corresponding range of transcribed genes runs only from 1 to 10^4^; i.e., protein abundance varies up to 1000 times more than RNA transcripts [71,72,73]. In fluids such as plasma, this variation is even wider—12 orders of magnitude [74]. Consequently, proteomics suffers from a restricted limit of detection (LOD) because it is still impossible to detect all proteins existing in a biological sample. Another problem is that a protein of interest cannot be amplified to the level of detection; therefore, their concentration levels in a given sample translate directly into the linear dynamic range required for its analytical method.

**Figure 6:**
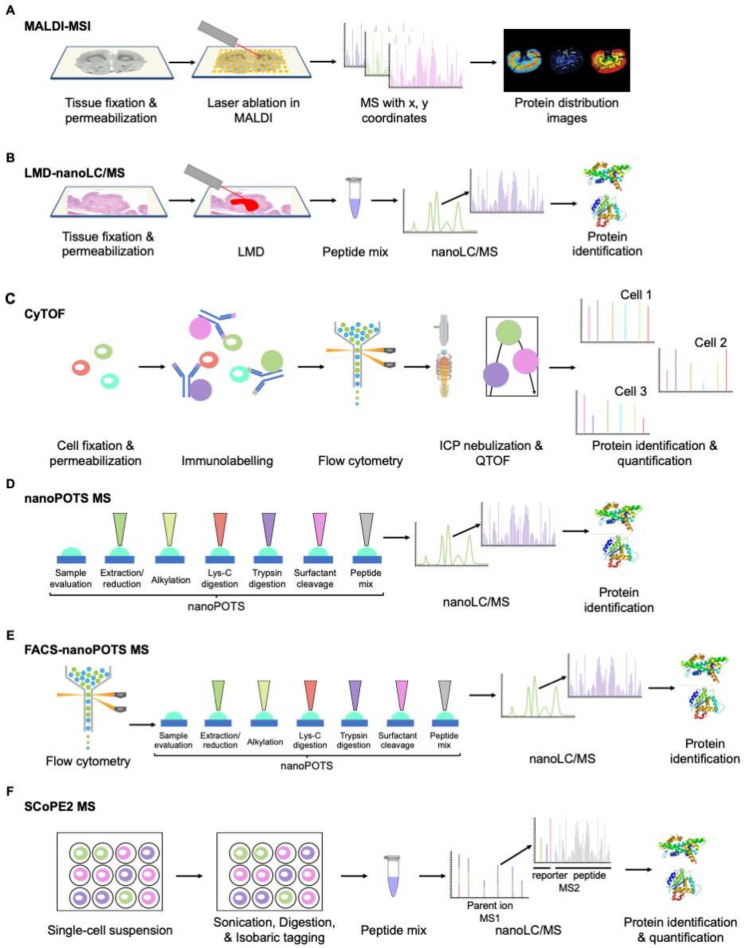
(**A**,**B**) Hyphenated mass spectrometry techniques orientated to the study of tissues. MALDI-MSI is a shotgun proteomics technique, whereas laser microdissection (LMD) selects a region of interest within the tissue and the laser capture for its subsequent analysis by nanoLC/MS. (**C**) CyTOF (mass cytometry) conjugates the potential of flow cytometry with the multiplexing capacity of mass spectrometry by using a pool of metal-labelled antibodies. Metal signals are analyzed by their time-of-flight and their relative intensity allows for protein quantification from each cell. (**D**–**F**) nanoPOTS (nanodroplet processing in One-pot for Trace Samples) and SCOPE2 MS (Single Cell ProtEomics by Mass Spectrometry) are microfluidic-based platforms that enable single-cell proteomics analysis and can even be hyphenated to other techniques, such as FACS (fluorescence-activated cell sorting) flow cytometry. With nanoPOTS the protein sample is processed onto individual spots, whereas SCOPE2 consists of a peptide isobaric tagging before mixing all the samples in a pot. The parent ion of interest is further analyzed by MS/MS and reporter ions serve to perform relative quantification.

In addition, mass spectrometry has traditionally been employed for analyzing in-a-pot, in-solution samples; i.e., sample preparation before its analysis on the MS platform implies extracting proteins from isolated cells or tissues, and subsequent chemical and enzymatic processing to generate a liquid dispersion of peptides suitable to inject into a separation/analytical platform in sufficient quantities to enable their identification and/or quantification. This scheme hampered the development of hyphenated techniques orientated to mine the proteome of tissues maintaining spatial topology, with the renowned exception of MALDI MSI (matrix-assisted laser desorption/ionization mass spectrometry imaging) (Figure 6A) [75,76].

Similarly, LMD (laser microdissection) and FACS (fluorescent-activated cell sorting)—as preparative techniques before mass spectrometry—are already feasible, although still complex (Figure 6B,E) [77,78]. Mass cytometry and microfluidics-based platforms (Figure 6C,D,F) represent an exciting and promising future dealing with the impossibility of amplifying the initial sample in a manner that genomics allows [79,80,81]. This scenario is in direct contrast to the situation over the last two decades, and it is worth pointing out that recent advances in single-cell proteomics dealing with the “world-to-chip” problem will aid in retaining spatial information in proteomics. In this sense, the progress of histoproteomics in the upcoming years should not be ignored [82,83].

## 4. Insights into the Histopathology of Bladder Cancer by Proteomics

Anatomically, the bladder has four sections: two inferolateral walls, the superior wall, or dome, and the posterior wall, or base, where the trigone locates. The latter is a triangular portion of smooth muscle that joins with the urethra in the bladder neck. It is histologically different because it controls the need for voiding; hence, it is highly innervated and very sensitive. In fact, whereas the rest of the bladder surface contracts and expands to allow the bladder to fill, thus modifying its thickness and folding, the trigone maintains a constant thickness and has a smooth surface. In addition, the trigone derives from a mesodermal germ layer, whereas the rest of the bladder comes from the endoderm, a key difference that allows researchers to suggest the utility of specific protein markers to identify the embryological cell origin within the bladder [84,85,86,87].

Lymphatic drainage, it is conducted through the external and internal iliac lymph nodes, with portions of the bladder neck draining towards the sacral and common iliac lymph nodes. Moreover, it is primarily vascularized from the internal iliac vessels, with the arterial supply running through the superior vesical branch, and venous drainage running through the vesical venous plexus. In contrast, the nervous supply is more complex; neurological control of the bladder is quite complex because this organ receives inputs from the autonomic and somatic systems. The hypogastric nerve conducts sympathetic reflexes, promoting relaxation of the detrusor, and the pelvic nerve conducts the parasympathetic reflexes, promoting the contraction of the detrusor; the pudendal nerve innervates the urethra and provides voluntary control over micturition. In addition, sensory nerves conduct the afferent stimulus to the brain.

**Figure 7:**
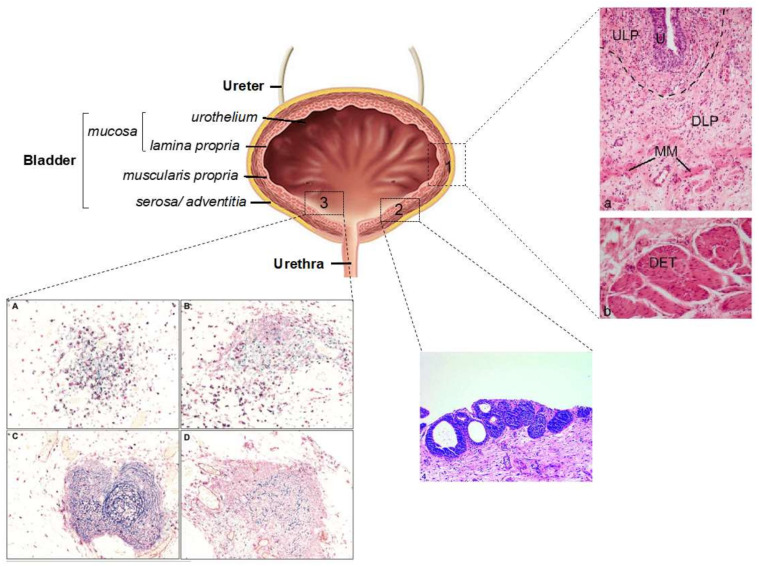
Schematic representation of the main sublayers of the bladder with an amplificated view to show a (**1**) Hematoxylin–eosin staining of the (**a**) mucosa of the bladder and (**b**) the *muscularis propria*. (U) refers to the urothelium, and the *lamina propria* (LP) is split between the upper *lamina propria* (ULP) and deeper *lamina propria* (DLP) with a dashed line, with the *muscularis mucosae* (MM) below and a separated view of the detrusor muscle (DET), located in the *muscularis propria* layer [88]. (**2**) Histological staining of von Brunn nests. (**3**) Multi-IHC view of bladder lymphoid aggregates from type 1 to type 4: CD4+ T cells (green), CD8+ T cells (purple), CD20+ B cells (pink), CD21+ and CD208+ follicular dendritic cells (blue and black, respectively) [89].

Histologically, the bladder is structured in four layers, from inside out: urothelium, or lining epithelium, *lamina propria*, *muscularis propria*, and serosa/adventitia (Figure 7). Furthermore, the urothelium and the *lamina propria* are named together as the bladder mucosa, although this is a misleading term because there are no mucus-secreting specialized cells.

The scope of this section is to outline the contributions of proteomics to the classification of BC, and to the quest for protein biomarkers related to the pathophysiology of bladder/urothelial cancer. Manuscripts from the past five years were reviewed, containing the following keywords: (i) “bladder” OR “urothelial”, AND “cancer”, AND “proteomics” OR “proteome”, AND “tissue” OR “histology”; or (ii) “histochem”, AND “urothelial” OR “bladder”, AND “cancer”. Specific proteins of potential interest were searched by combining the protein name, AND “urothelial” OR “bladder”, AND “cancer”.

### 4.1. Recent Contributions to the Proteomics of the Urothelium

The lining epithelium, or urothelium, is a specialized transitional epithelium which not only coats the bladder, but the renal calyces, the renal pelvis, the ureters, and the urethra; succinctly, the structures that compose the urinary tract are in direct contact with urine. The urothelium is layered into the apical layer, the intermediate layer, and the basal layer. As was previously described, BC can be divided into two molecular subtypes, referred to as luminal and basal, the former mimicking the protein profile of the apical layer, and the latter resembling the proteome of intermediate/basal layers [90].

The apical layer is the innermost layer, and it serves as a protective barrier against the urine. It is composed of umbrella-shaped cells forming a tight-junction barrier covered by a scalloped layer of plaques formed by urothelium-specific transmembrane proteins known as uroplakins (UPKs) that are connected to the cell cytoplasm by actin filaments [91,92,93,94,95]. UPKs are markers of terminal urothelial differentiation and consist of four isoforms: UPK-Ia, UPK-Ib, UPK-II, and UPK-IIIa. Of these, UPK-Ia, and UPK-II have attracted attention for being tissue-specific, and having higher sensitivity and specificity to study bladder tissue sections by immunohistochemistry [96,97,98].

Umbrella cells are also responsible for the secretion of mucins, primarily MUC1, but MUC4 and MUC3 have also been detected [99]. Other mucins, such as MUC2, MUC6, and MUC7, or the syalomucin CD164 are specific to cancerous cells [100,101,102,103]. However, mucins such as MUC1 or MUC4 have been detected in both tumoral and healthy tissues, differing in the cellular space where they are expressed or, more frequently, by their expression levels [101,102,104]. Consequently, their adoption as clinical biomarkers has been hampered. In this regard, the behavior of MUC4 is exemplary, because it is mainly expressed by healthy cells, almost becoming lost in BC, but then reappearing in a subset of metastatic BC cases with worse outcomes [102]. Notably, we hypothesize that this oscillating expression of MUC4 during tumoral progression may be useful for monitoring cancer relapse with increased aggressiveness, especially if we notice that a significant number of NMIBC cases will evolve during the patients’ lifetime.

In addition to uroplakins and mucins, other protein markers of the apical layer/luminal tumors are GATA3 (trans-acting T-cell-specific transcription factor), Src (proto-oncogene tyrosine-protein kinase Src), FAK (focal adhesion kinase), CCND1 (cyclin-D1), CDHE (E-cadherin), KRT18 (keratin-18), KRT20 (keratin-20), and HER2 (receptor tyrosine-protein kinase erbB-2).

GATA3 is a zinc finger transcription factor that binds to the enhancer of the *TRAC* and *CD3D* (T-cell receptor alpha, and delta, respectively) genes, then contributes to T-cell development and differentiation, and regulating the proliferation and differentiation of nonhematopoietic cell lineages. Although GATA3 expression levels are increased in luminal BC tumors, their molecular mechanisms are not fully understood. A recent publication described its implication in arresting the cell cycle in G2/M and S phases, thus inhibiting cell proliferation [105]. This could also explain why patients in the initial stages of the disease with GATA+ overexpression had better outcomes [105].

Src, as a non-receptor tyrosine kinase, phosphorylates specific residues in other tyrosine kinases and participates in the regulation of cell development and proliferation. Recently, Src has been uncovered to play an additional role as a key mediator in the function of FAK, another non-receptor tyrosine kinase activated by extracellular signals such as some growth factors and integrins [106]. Consequentiy, Src is involved in cell motility. FAK and Src phosphorylation is mutually interdependent, and they both affect cell motility; therefore a potential role promoting the invasion and proliferation of cancer cells will be surprising. In fact, it has been demonstrated that patients diagnosed with hypertrophic scar condition, a rare disease associated with high levels of these kinases, benefited from targeted therapy [107]. Interestingly, inhibitors of these kinases showed promising results in breast cancer [108,109], a cancer type that shares common qualities with BC.

CCND1 functions by forming a protein complex with cyclin-dependent kinases CDK4 or CDK6, which is necessary for overpassing the cell-cycle G1/S transition breakpoint. During the G1 phase, it is synthesized and accumulates in the nucleus, and is rapidly degraded as the cell enters the S phase. As CCND1 participates in cell division, and proliferation, it can contribute to tumorigenesis. However, elevations in CCND1 expression levels have been detected in several types of cancer, although its relationship with outcome sremains obscure, having no correlation in BC [110]. However, it is frequently overexpressed in luminal tumors [111]; thus, it is plausible that CCND1 may play a role in tumor recurrence, a quite common event in bladder cancer.

Cadherins are a group of proteins responsible for cell–cell adhesion in epithelial cells; thus, their expression is expected to occur in the urothelium. Moreover, the abnormal expression of cadherins is a common feature in cancer, with the well-known cadherin switching event promoting a loss of E-cadherin and an overexpression of N- or P-cadherins. This cadherin switching has previously been detected in BC as well as other types of tumors and is characteristic of tumoral progression and worsening [112]. Additionally, there is close crosstalk between the cadherin-switching episode and EMT (epithelial–mesenchymal transition), a crucial event in tumorigenesis, enhanced metastasis, chemoresistance, and tumor stemness. As noted, the molecular subtyping of BC tumors identified a subset of BC tumors expressing aberrant levels of CAMs (cell adhesion molecules), specifically, the claudin-low subtype [33]. This subtype was associated with an enhanced EMT, hence having a high-grade, muscle-invasive status. Due to this mutual interdependence, it would be expected that the dysregulation of CAMs such as E-cadherin, claudins or even TJP1 (tight junction protein 1) would promote EMT. However, it has been shown that a positive expression of E-cadherin in bladder cancer cells is the key underlying feature of the intraepithelial expansion of the tumor [113]. Consequently, we cannot simplify the tumoral progression focusing solely on the presence/absence of EMT. This framework explains the recent work of Guo, C. et al. [114]. By analyzing the expression of a gene panel in luminal and basal cancers, they conceived the basal to luminal transition (BLT) score as a quantitative classifier that offers great sensitivity and specificity and promising clinical utility. Intriguingly, although the luminal subtype had a positive BLT score, and a positive EMT score, the basal subtype had a negative BLT score, and a positive EMT score. Within the EMT panel, the authors included the *CDH1*, *CLDN1*, and *TJP1* gene transcripts. In conclusion, the role of CAMs and their relationship with the cell dynamics in EMT and tumoral progression in BC should be further explored and defined.

KRT18 and 20 are two cytokeratins that define the luminal subtype, later stages of differentiation of urothelial cells, or, inversely, early stages of BC (Figure 2B) [22,26]. Cytokeratins are an extensive family of proteins expressed by epithelial and mesothelial cells, and linked with the intermediate filaments; hence, their presence is expected in the urothelium. Of the two, KRT20 has broadly been used as an immunochemical marker because it is predominantly expressed by superficial, well-differentiated umbrella cells. Dysregulation by overexpression in KRT20 expression is observed in several histological types of BC [115,116], and its determination is endorsed by the guidelines of the International Society of Urologic Pathology (ISUP) and the European Network of Uropathology (ENUP) [117,118]. Recently, the prognostic potential of KRT20 and KRT5 has been tested by mRNA expression in a cohort of 122 patients, where high expression levels of *KRT5* and low expression levels of *KRT20* were associated with a significantly better outcome measured by the recurrence-free survival (RFS) and disease-specific survival (DSS) rates [119]. The authors also found a correlation between outcomes and the immunochemical expression of both cytokeratins. This research agrees with previous reports that found KRT20 overexpression to be an important biomarker of tumor recurrence, in comparison with lower expression levels in the normal urothelium [120,121,122]. Interestingly, these researchers pointed out the importance of not describing the tumor grade or stage alone. Although BC tumors with KRT20 overexpression are not suspected of being as menacing as the aggressive KRT5+ MIBC tumors, there is also strong evidence that they can exhibit increased rates of recurrence and tumor progression. Most importantly, the determination of *KRT20* by RT-PCR in cytology supports its use as a potential biomarker in liquid biopsies, although it remains to be investigated [123].

**Figure 8:**
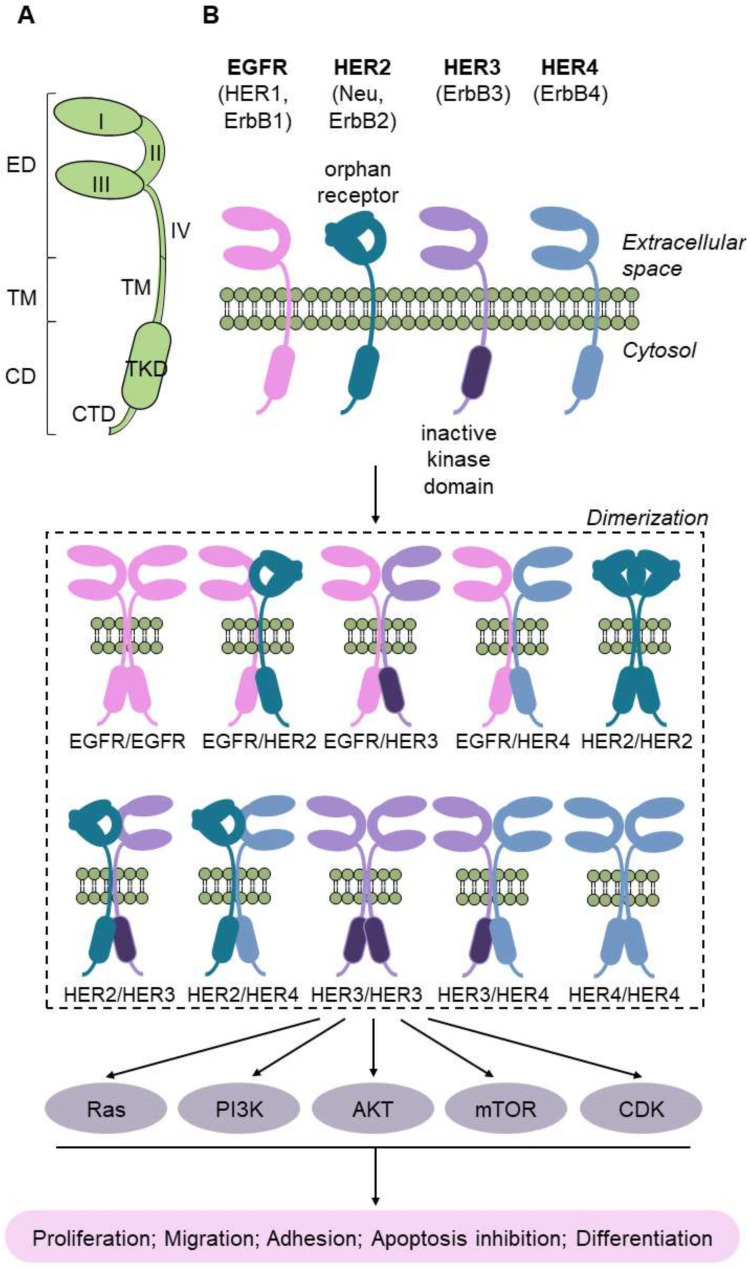
(**A**) Schematic representation of the three domains of an ErbB receptor. The N-terminal extracellular domain (ED) has four subdomains, with the subdomains I and II participating in ligand binding, and the II and IV subdomains participating of the receptor. TM refers to the transmembrane domain, and the cytoplasmatic domain (CD) has two subdomains more, with the tyrosine-kinase (TKD) and the C-terminal (CTD) subdomains. (**B**) Schematic representation of the 4 HER receptors and the 10 possible homo- and heterodimers. Activated HER dimers promote many signaling cascades affecting multiple key biological pathways.

The ErbB family is a class of receptor tyrosine kinases and comprises EGFR (HER1), ErrB2 (HER2), ErbB3 (HER3), and ErbB4 (HER4) (Figure 8). Upon ligand binding for HER1, HER3, and HER4, they dimerize, and this event activates downstream growth-signaling pathways. HER2, in contrast, is constitutively active and it has been demonstrated to participate in modulating cell proliferation and transformation by coactivation of the RAS/MAPK, PI3K/AKT, and JAK2/STAT3 signaling pathways during chemical insults [124,125]. This may suggest that HER2 bears a role in maintaining the integrity of the urothelium. In fact, mutations in *HER2* are commonly found in BC, with an aberrant overexpression of the corresponding protein by immunohistochemistry [126]. EGFR and HER2 overexpression are associated with worse tumor grade and invasiveness, recurrence, metastasis, and shorter overall survival [127]. As a biomarker, HER2 overexpression was higher in later-stage patients, with the particularity that they were potential responders to afatinib [128,129].

The intermediate layer is stratified by two or three layers of polygonal columnar cells, whereas in the basal layer, there are small cuboidal cells attached to the base membrane with hemidesmosomes. Within these layers, there is a subpopulation of KRT5+ (keratin-5) intermediate cells responsible for urothelium regeneration and reparation after insults [130,131]. A more primitive KRT14+ (keratin-14) cell population has also been found and participates in cell proliferation [26,132]. More importantly, it has been shown that KRT5+ and KRT14+ keratinocytes within the intermediate and basal layers responded to insults in a complementary way [133]. Although KRT5+ cells proliferated in the initial steps in response to the presence of KGF (keratinocyte growth factor), the proliferation of KRT14+ keratinocytes was unaffected by KGF and occurred at a later stage. This is an important finding that may explain tumorigenesis in the urothelium as deregulation of the equilibrium in the proliferation of this cell population [134]. In addition, it has been found that keratinocyte populations within the intermediate and basal layers can be selectively enriched after successive chemotherapy cycles [135], and this may explain why a basal subtype of BC has been described as more aggressive, with shorter survival, although greater sensitivity to cisplatinum-based chemotherapy [46]. Instead of focusing on short-term positive responses, novel treatments should be based on affecting the ratio of these cell populations, and proteomics represents a favorable tool for selectively monitoring these changes in response to the proposed therapy.

Recalling the cadherin-switching event, overexpression of P-cadherin, also known as CDH3, is considered a characteristic hallmark of the basal urothelial cancer subtype. However, its expression is localized in a single-cell layer along the *lamina propria* [46]. Less is known about its prognostic value in BC, because the role of P-cadherin in the normal epithelium is unidentified, and its role in cancer depends on the disease type [136]. In a study conducted with 110 NMIBC cases, the overexpression of P-cadherin was suggested as a predictor of BC progression [137]. In line with this finding, an earlier study with RT-112 cells from BC showed that stable attenuation in the expression of P-cadherin diminished the invasion and migration rates of these cells, whereas the cell-to-cell adhesion was enhanced [136]. However, the determination of P-cadherin in 536 BC paraffined sections by Mandeville, J.A. et al. showed that a lower expression of this protein was linked with a worse outcome in patients [138]. In contrast, when the authors forced the expression of this protein in EJ and UM-UC-3 cells—two BC cell lines that constitutively lack P-cadherin expression—both cell lines acquired an enhanced migration rate [138]. These opposing results were explained by the relocation of P-cadherin, because patients—where this protein concentrates in the cytoplasm—exhibited a shorter cancer-specific survival (CSS) rate than those where the expression of P-cadherin was circumscribed to the cell membrane. Other work related to the cadherin-switch of basal cells was published by Genitsch, V. et al. with a cohort of 21 MIBC patients [139]. They identified a shift between the molecular subtypes at the transcriptome level, and evidence of active EMT from urothelial toward a sarcomatoid morphology by analyzing the expression of E-cadherin, Zeb1, and TWIST1.

### 4.2. Recent Contributions to the Proteomics of the Lamina Propria

The *lamina propria* is between the urothelium and the deeper *muscularis propria*, and includes a thin smooth muscle sublayer, the *muscularis mucosae*. In the trigone, it is thinner than in the bladder body. Elastic protein fibers are embedded in an extracellular matrix with a high proliferation of immune cells, fibroblasts, adipocytes, and interstitial cells such as telocytes, Cajal cells and other lineages [140]. Gabella, G. et al. used the bladder of adult rats as a model to describe an internal substructure of the *lamina propria*, consisting of two sections: the most superficial layer, adjacent to the urothelium, is rich in fibroblasts, nerves, small blood vessels, and with thin collagen bunches; the deepest layer is more fibrous and, thus, richer in collagen, with a lower fibroblast population [141]. This is in agreement with previous reports that also found differences in the interstitial cell phenotype depending on its layer position inside the mucosa [142]. In addition, the *lamina propria* also contains an intertwined network of vascular and lymphatic vessels, afferent and efferent nerve endings, and indistinct smooth muscle fascicles and the *muscularis mucosae* that conforms the detrusor muscle [143]. Thus, the bladder *lamina propria* can be considered a hub to integrate and transduce signals from and towards the immune, nervous, vascular, and muscular systems. However, despite being the transitional boundary from the mucosa to the muscular layer (and, therefore, the field of such a complex signaling system [144]), the urothelium has received much more attention, whereas the cellular histology and physiology of the *lamina propria* remains elusive.

As a paradigmatic example, we can cite the identification of interstitial Cajal cells. Unlike the interstitial Cajal cells found in the gut, their role and presence in the bladder *lamina propria* is still not well understood and they have often been misidentified as other cell lineages. They are believed to function as intermediary pacemakers by modulating the effect of neurotransmitters onto the adjacent smooth muscle cells [145]; hence, their stellate shape with long dendrites has allowed them to establish contact with nerves and muscle cells by providing an intercellular signaling route. Thus, the integrity of the gap junctions may be critical to their function [146], an idea that leads to the investigation and further recruitment of proteins such as c-Kit (CD117), hematopoietic progenitor cell antigen CD34, Anoctamin 1 (Ano1), ectonucleoside triphosphate diphosphohydrolase 2 (NTPDase2), connexin-43, vimentin, desmin, PDGFβR, and merlin (NF2) as specific biomarkers for interstitial Cajal cells, differentiating them from myocytes and myofibroblasts [147]. Despite this assumption, the employment of c-Kit as the “gold standard” biomarker for identifying this cell subpopulation, as perfomed in the gastrointestinal tract, is still suspicious and should be used with caution. Unlike in the gut, c-Kit+ cells in human and other mammal bladder tissues could not be univocally identified as interstitial Cajal cells, but myeloid lineage-derived mast cells [88,148]. This finding would explain some discrepancies published in the early 2000s [149,150,151]. Furthermore, other authors proposed that CD34+/c-kit cell populations should be identified as interstitial Cajal-like cells (ICLCs) [152], whereas CD34+/PDGFRα+ cells should be classified as telocytes [153]. Importantly, ample debate persists regarding the classification of these urinary interstitial cells. However, their characterization seems important, because in the rat model these cells responded to the presence of cyclophosphamide by triggering hyperplasia and hypertrophy [154]. In contrast, prior treatment with imatinib prevented their proliferation, probably because of the affectation of endothelial NO synthase activity [154], which is regulated by phosphorylation.

Within the *lamina propria*, von Brunn’s nests are benign encapsulations of urothelial-like cells whose appearance mimics a rare urothelium tumor variant known as nested urothelium carcinoma (Figure 7) [155]. It is thought that 89% of grossly normal bladders exhibit von Brunn’s nests; hence, their presence cannot be ascribed to a malignant tumor [156]. However, the origin of von Brunn’s nests has not yet been elucidated, and two models have been proposed: either a direct invagination of healthy urothelial cells, probably due to defects in the cell-to-cell and cell-to-ECM junctions, or differentiation from another cell type. Histologically, von Brunn’s nests are stratified, polarized urothelial-like cells enveloped in a capsule enriched in Collagen-I, -III, and -IV. In addition, these cells show phenotypical changes compared to cells from the urothelium, such as the expression of FGF10 receptors, or cytokeratins, such as KRT7 and KRT13 [157]. These results support the idea that von Brunn’s nests may derive from subpopulations already located in the *lamina propria*, such as 10pRp cells [157]. Moreover, a recent phylogenetic study pointed to a single clone origin, with all cells within a nest derived from a single parent [158].

Lymphoid aggregates are common in the *lamina propria*, and they reflect the adaptative response of these tertiary immune organs to tumoral growth and inflammation. Type 1 aggregates are compact, small, and with an abundance of T-cells; type 2 aggregates differ from type 1 in that they are larger; type 3 aggregates have a core of T-cells surrounded by germinal centers of B-cells and follicular dendritic cells; and type 4 aggregates almost lack this core of T-cells, consisting of B-cells and follicular dendritic cells. Interestingly, changes in the lymphoid aggregates are a common event in aggressive BC [89].

A recent study conducted by Koshkin, V.S. et al. lucidated the role played by DLL3 (delta-like ligand 3) in small-cell BC, a rare neuroendocrine BC subtype [159]. This protein is an inhibitory ligand of the Notch pathway and was found to be upregulated on the cell surface in small-cell lung cancer and other neuroendocrine tumors [160]. The Notch pathway is a highly conserved cell-cell signaling pathway that participates in cell processes such as angiogenesis, lymphocyte expansion, and the promotion of proliferative signaling during neurogenesis. Its activation is triggered via direct cell-to-cell contact after DLL3 interaction with the NOTCH2 receptor and subsequent release of the intracellular domain, NICD2, which translocates into the cell nucleus to act as a gene transcription regulator. Conversely, DLL3 expression levels are affected by the transcription factor achaete-scute homolog 1 (ASCL1). When Koshkin V.S. et al. measured the protein expression levels of DLL3, ASCL1, PD-L1 and CD56 by immunohistochemistry, they found that DLL3 and PD-L1 were prognostic biomarkers that correlated with shorter OS [159]. An interdependence between cancer cells and the immune system can be established through DLL3, because high DLL3 expression enables the development of targeted immune-oncological therapeutics schemas, such as the antibody–drug conjugate rovalpituzumab tesirine, the bi-specific T-cell engager (BiTE^®^) AMG 757, or the chimeric antigen receptor T-cell (CAR-T) therapy AMG 119. All of these therapeutics have been investigated in small-cell lung cancer; however, their utility in small-cell BC cannot be neglected [161].

**Figure 9:**
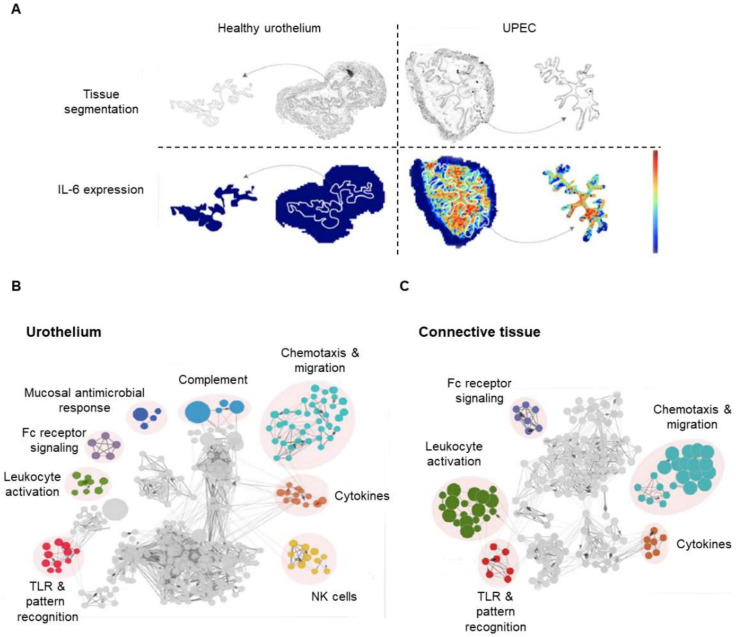
Schemes follow the same formatting. (**A**) Representative spatial distribution of IL-6 in healthy and infected (UPEC) bladder tissue by MALDI-MSI. (**B**,**C**) Enrichment functional analysis of the significantly upregulated proteins after infection in the urothelium by interaction networks (**B**) or the connective tissue (**C**) [162].

After damage, crosstalk communication between macrophages and cells of the urothelium stimulates the migration of the former cells towards the urothelium. In a novel experiment, it was demonstrated, through MALDI-MSI, that the molecular mechanisms involved in the migration of macrophages increase upon bladder infection (Figure 9A) [162]. MALDI-MSI, and an enrichment analysis by Cytoscape/ClueGO, identified the upregulation of proteins participating in leukocyte activation, cell migration, cytokine production, and activation of the FcR signaling and the recognition of Toll-like receptors (TLR) (Figure 9B,C). Briefly, macrophage chemotaxis and the subsequent activation of the immune response was mainly mediated by IL-6 and protease-dependent mesenchymal migration. In contrast, the lack of IL-6 corresponded with decreased levels of the metalloproteinases MMP-2, -9, and -28. Inhibition of IL-6 also caused a decrease in CX3CL1 signaling. CX3CL1, also known as fractalkine, is an inflammatory chemokine with the single receptor CX3CR1 which regulates cytotoxic T cell-mediated immunity by recruiting TIL (tumor-infiltrating lymphocytes) to the tumoral microenvironment [163]. When expressed by cancerous cells, it promotes migration and invasion [164]. However, further research is needed to elucidate the role of fractalkine in BC. More is known about IL-16, a cytokine that has been demonstrated to be seminal in regulating the sentinel node-Treg cell signaling network [165]. Using a proteomic approach, the authors demonstrated that Treg cells were able to process IL-16 in response to the presence of tumor-released factors, with the final consequence of reinforcing the suppressive role of Treg cells, triggering an enhanced immune evasion of cancerous cells.

### 4.3. Recent Contributions to the Proteomics of the Muscularis Propria

The *muscularis propria*, or detrusor muscle, consists of smooth muscle. It has three sublayers: the inner and outer layers made up of longitudinal fibers, and circular fibers in the middle layer. However, these layers are loosely defined, and they are randomly disposed of, except for the bladder neck. Furthermore, they have fewer smooth muscle cells and higher connective tissue in the trigone than in the dome, which suggests the existence of a structured network of myocytes and myofibroblasts [166]. These differences between the *muscularis propria* of the dome and trigone were further assayed by measuring the mRNA expression levels of a panel of 20 transcripts, with further analysis of the expression levels of the tachykinin receptor-1 (NK1R), occluding (OCLN), and acid-sensing channel ASIC1 by immunofluorescence, with the smooth muscle actin (SMA) marker for discriminating muscle cells [167]. Higher levels of spontaneous activity, smaller myocytes and differences in the gap junction protein expression were also observed in trigone *muscularis propria* from a guinea pig model [168]. Interestingly, the utility of smoothelin in an immunohistochemistry analysis was demonstrated to be higher than the more common SMA marker in order to discriminate the *muscularis propria* from the *muscularis mucosae*, an important feature to evaluate more precisely the advancement of bladder cancer through the bladder [169]. Briefly, although SMA was expressed by myocites and myofibroblasts, smoothelin was particularly present in well-differentiated smooth muscle myocites [170]. Despite these early contributions, further experiments were unable to support the use of smoothelin as a reliable marker for staging BC by immunochemistry methods, although this was further discussed due to a lack of optimization of the immunostaining protocols [171,172,173,174]. The utility of SMA and smoothelin in evaluating the invasion of the muscular layer in BC should be considered in future proteomic approaches, especially because of the great interest it would have to stage the lesion more precisely.

### 4.4. Recent Contributions to the Proteomics of the Serosa Adventitia

The serosa layer is, essentially, connective tissue partially covered with a mesothelial lining at the peritoneal area. It is a thin, loose layer that can barely prevent the spread of tumor growth, and as it is vascularized—with nearby lymph nodes surrounded by perivesical fat—its invasion presents a great risk to which leads metastasis [175,176]. With advancing age or obesity, serosal fat permeates towards the *muscularis propria* and even the *lamina propria*, and it has been reported that adipose stromal cells present in this fat are a relevant source of secreted cytokines and chemokines such as IL-6 whose expression exerts mitogenic effects in cancer [177,178,179,180]. Using rat models, immune mast cells have been identified in this layer [181]. Moreover, Cav-1 (caveolin-1) was abundantly expressed in the serosa, whereas Cav-2 and Cav-3 were not [182]. Cav-1 and Cav-3 can drive the formation of caveolae, an invagination of the cell membrane during the endocytic pathway, acting as scaffolding proteins. Moreover, Cav-1 is also associated with DPP4, CTNNB1 and TGFBR1 [183,184,185]. Similarly, Cav-3 is also involved in regulating the GPCR signaling pathway mediated by adenylyl cyclase [186]. In contrast, Cav-2 influences the formation of caveolae by triggering the caveolin-1-mediated pathway and also lessens the inhibiting effects of Cav-1 onto the nerve growth factor (NGF) pathway and subsequent cell differentiation [187,188]. Moreover, whereas the expression of Cav-3 is circumscribed to muscle cells, the expression of Cav-1 and Cav-2 identifies the presence of adipocytes, fibroblasts, and other endothelial cells. Taken together, as mediators of endocytosis and related to cell signaling, caveolins are implicated in both carcinogenesis and tumoral suppression [189], and they probably act as promoters in the case of malignant bladder tumors [62,190]. However, and despite the potential interest that proteins found in the serosa may have, the tumoral lesions arising in this bladder layer are exceedingly rare [191,192]; thus, the influence of this molecular environment in BC is largely unknown [193,194,195].

### 4.5. The Dawn of the Histoproteomics of Bladder Cancer

An interesting work published in 2015 by Habuka, M. et al. employed RNA-Seq to identify genes that were upregulated in the bladder compared with a pool of tissues—a classification complemented by localizing the expressed proteins by immunohistochemistry and tissue microarray techniques [196]. Ninety protein-coding genes were identified, with the four uroplakins (*UPK1A*, *2*, *3A*, and *3B*) appearing solely in the umbrella cells, three in the intermediate/basal cells (*KRT17*, *PCP4L1*, and *ATP1A4*), and twenty in the entire urothelium (*CLEC3A*, *DHRS2*, *HOXA1*, *PADI3*, *DUOXA2*, *BMP3*, *RBFOX3*, *ACER2*, *CCR8*, *CYP1A1*, *PTGS2*, *OSTN*, *IL24*, *MYO3B*, *CTHRC1*, *CHRDL2*, and *UPK1B*). Interestingly, this study relates the information gathered by genomics and proteomics in a single set of data, linking them with the spatial structure of the bladder tissue.

Advances over this basic schema have also been published. Witzke, K.E. et al., investigated histoproteomics of CIS by label-free Fourier transform infrared (FTIR)-guided laser microdissection (LMD) of the region of interest and a hyphenated LC-MS analysis (scheme in Figure 6B) [197]. They identified three potential biomarkers from a total set of almost 2500 proteins, AHNAK2 being the most promising. The function of AHNAK2 remains unresolved, although it is believed to participate in calcium signaling by interacting with calcium channel proteins. Its overexpression has also been identified in renal, lung and pancreatic cancers, and knockdown of AHNAK2 inhibited the proliferation, colony formation and migration in vitro and in vivo tumorigenic ability of cancer cells [198,199]. Another study, conducted by Lee, H. et al., identified AHNAK (neuroblast differentiation-associated protein AHNAK) as a potential biomarker of BC from a total of 4839 proteins identified by LC-MS, together with EPPK1, MUYH14, and OLFM4 [200]. Most interestingly, AHNAK was the only protein whose expression was further detected by liquid-based cytology. Moreover, previous reports described the tumor suppressing role of AHNAK by influencing E-cadherin expression, and the TGFβ/Smad signaling pathway [201,202]. Taken together, these studies are of utmost importance not because of investigating the AHNAK family, but because, as far as we know, these are the first reports that link data from genomics to those obtained by spatial proteomics, which also validated these protein biomarkers identified in tissue biopsies by liquid biopsy.

In this regard, the importance of liquid biopsy is not only to offer an easy sampling protocol but to also enable the detection of specific secreted proteins. In line with this, Ho M.E. et al. investigated the expression of AGR2 (anterior gradient 2) by BC cells [203]. This protein is also expressed by a broad representation of cancer cells, but normal cells also express AGR2. However, the concomitant presence of AGR2 in urine and its localization to the cell surface was a characteristic feature of BC cells, whereas the expression of AGR2 onto the cells by itself was not. Additionally, proteomics analysis of the blood contained no significant AGR2 levels, suggesting that there is minor urothelial secretion through the capillaries of the *lamina propria*.

In 2014, MALDI-MSI was investigated as a promising tool to predict the progression of NMBIC to more aggressive stages (Figure 6A) [204]. A study of paraffined tissue sections from 697 patients using this technique revealed 40 *m*/*z* signals associated with tumor aggressiveness (30 signals), in situ or papillary growth (5 and 3 signals, respectively), and increased or decreased cell proliferation (6 and 12 signals, respectively). The possibility of predicting recurrence in a non-invasive Ta stage or by progressing to a more advanced stage was also feasible (2 signals at *m*/*z* = 775.9 and 2705.8). In addition, the absence of a characteristic signal at *m*/*z* = 701.9 concurred with a decreased survival rate in MIBC cases. However, despite the promising prognostic capabilities of MALDI-MSI in BC, only eight signals could be identified by MS2, namely the histone H2AC1, cytokeratins KRT19 and KRT7, hemoglobin, collagen-I alpha chain, and HSPB1 (heat-shock protein beta-1), which was a limitation of this intriguing study because none of the signals could be ascribed to recurrence/progression, with the sole exception of the signal at *m*/*z* = 2705.8, corresponding to a peptide from the collagen-I alpha chain. This collagen isoform was described as part of the matrix capsule of von Brunn’s nests, as noted earlier, although a recent publication also identified an aberrant expression of collagen-I during NMIBC progression with poor prognosis in a cohort of 189 patients [205]. Mechanistically, overexpression of collagen-I in BC remains elusive, although it points to a key role in the remodeling of the interstitial matrix of the ECM [206]. Patients with a high expression of collagen-I in the tumor-ECM boundary showed a worse progression of BC in comparison with the other three patterns (expression within the thin linings of the stroma, expression within the stroma vasculature, expression surrounding epithelial tumor cells, and expression within the *lamina propria* near the tumor-ECM boundary) [205].

The potential of MALDI-MSI can even be developed in terms of localizing a broad scale of proteins. A serious limitation in using a single tissue section is the restraint regarding tumor heterogeneity by studying it from a bi-dimensional approach. Considering that histoproteomics offers invaluable advantages to cope with spatial features as well as large numbers of proteins, the development of methods able to cope with the tridimensionality of the tumors would be a key milestone. As a theoretical concept, maintaining the *m*/*z* information within the corresponding *x,y,z* coordinates is technically challenging, with the increased risk of introducing biases into the data. In this regard, one recent study published in 2019 investigated the capabilities of 3D MALDI-MSI in BC [207]. By analyzing 20 consecutive slice sections of 14 tumors, the authors could identify the outliers in a peak list of almost 300 signals by two methods: cytochrome c digestion to monitor the efficacy of the enzymatic digestion, and the z-directed distribution of the *m*/*z* intensity using a regression method. When these two criteria were applied, they demonstrated increased robustness in the consistency of the spatial correlation, and accurate visualization of the tumor sample, both features facilitating the discovery of protein biomarkers.

## 5. Proteomics in the Therapy of Bladder Cancer

**Figure 10:**
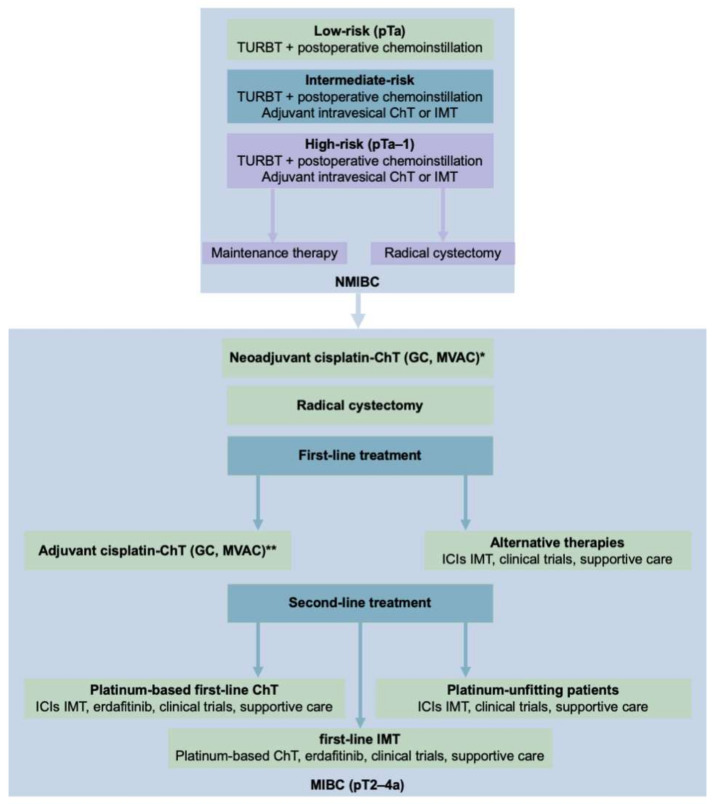
Schematic representation of the therapeutical management algorithm for bladder cancer. TURBT: transurethral resection of bladder tumor; ChT: chemotherapy; IMT: immunotherapy; GC: gemcitabine/cisplatin; MVAC: methotrexate/vinblastine/doxorubicin/cisplatin; NMIBC: non-muscle-invasive bladder cancer; MIBC: muscle-invasive bladder cancer; ICIs: immune checkpoint inhibitors. *: optative for platinum-based ChT fitting-patients. **: offered if no neoadjuvant ChT used.

In BC, a conjunction of systemic and local therapies is the conventional approach, with the transurethral resection of bladder tumor (TURBT) being the typical surgery. In NMIBC, the postoperative prescription of chemo-instillation is highly recommended after TURBT, with adjuvant intravesical instillation with either mitomycin C chemotherapy or Bacillus Calmette-Guérin (BCG) immunotherapy in intermediate- and high-grade tumors. Non-responders are usually subjected to radical cystectomy (Figure 10).

In contrast, there are great differences in the treatment scheme followed in MIBC. For BC patients primarily diagnosed with MIBC, as well as in non-responding and progressing NMIBC cases, radical cystectomy is the recommended surgery, with the possible removal of ureters and prostate and seminal vesicles in men, or uterus and part of the vagina in women. Neoadjuvant or adjuvant platinum chemotherapy is also customarily offered (Figure 10). Currently, there is a diverse range of dosage schemes are being tested in different clinical trials [208]. If metastasis is present at the moment of diagnosis or after relapse, platinum-based chemotherapy is also the palliative treatment. Two combinations are regularly used: methotrexate/vinblastine/doxorubicin/cisplatin (MVAC) or gemcitabine/cisplatin (GC), with the optional prescription of granulocyte colony-stimulating factor (G-CSF) to facilitate the recovery of bone marrow between cycles [208].

### 5.1. Response to Platinum-Based Therapies in Bladder Cancer

However, the benefits of platinum-based therapies are still limited to a subset of patients and are unpredictable due to the current absence of valid biomarkers. Platinum conjugates crosslink with DNA, affecting replication and transcription; thus, they force the activation of DNA repair pathways. Therefore, it is expected that an affectation of some participating genes would predict a positive response to platinum-based therapies. Following this hypothesis, van Allen, E.M. et al. found that mutations in *ERCC2* correlated with a good response of MIBC to cisplatin [209]. Similarly, patients harboring mutations in *ATM*, *RB1*, and *FANCC* were sensitive to platinum-based therapies, with *ERCC2* demonstrating its utility in a new cohort [210,211].

Interestingly, there are some features related to this panel of mutations that may predict the response of patients in the early stages of the disease, although it would require further investigation. For example, because *FANCC* is located in chromosome 9q, there could be a good response of NMIBC patients within the GS2 subgroup to platinum-based treatments, as proposed by Hurst, C.D. et al. [32]. In addition, it has been shown that mutations in *ERCC2* differentiate between the worse responses to platinum-based treatments of secondary MIBC cases (patients that progressed from an initial NMIBC diagnosis) and primary MIBC [8]. In line with this, the relationship between mutations in *RXRA* with differential responses and progression of NMIBC to MIBC should be further investigated [212].

Despite these promising findings, to date, almost no studies have employed a proteomics approach. Nevertheless, a recent publication has explored the major affectations arising in response to recurrent chemoradiation of a patient diagnosed with BC where the authors found that proteins related to the regulation of the BUB1B/BUBR1 mitotic checkpoint and chromosome segregation were clearly affected [213]. Of note, this finding was further validated in cell assays, demonstrating that resistance to chemoradiation arose in conjunction with an increased mutagenic rate in the non-homologous end-joining repair (NHEJ) of the double-strand breaks caused by the treatment itself. In addition, the restoration of cell sensitivity to cisplatin was feasible after attenuating the expression of these proteins. Briefly, a phenotypic selection by upregulating BUB1B/BUBR1 promoted the proliferation of chemoradioresistant cancerous cells with an increased mutation burden.

In addition, activating mutations in *ERBB2* also correlated with a good response to cisplatin [214]. As stated earlier, the corresponding protein HER2 is constitutively active and participates in cell proliferation and cell transformation in response to chemical insults [124,125]. Its its overexpression evades the stress signals triggered by the DNA damage response (DDR) pathways; thus, BC subtypes, as well as other HER2+ cancers such as breast cancer, have a significantly poorer prognosis and more aggressive behavior. However, their response to platinum-based chemotherapy, as demonstrated, is also better in BC than when *ERBB2* is unaffected [213]. However, only 23.6% of the responding patients harbored mutations in *ERBB2*, and the role of this molecular signature remains controversial [215].

Again, contributions to elucidate the functions of *ERBB2*/HER2 in BC using proteomics field are quite vague. An ambitious study was conducted which combined isobaric tag for relative and absolute quantitation-based mass spectrometry (iTRAQ-based MS) and reverse-phase proteomic array (RPPA) and provided a dataset of 331 unique protein candidates [216]. However, the results only supported the utility of HER2 as a promising target in the treatment of BC, and no information regarding the potentially affected pathways was provided. This contrasts with the numerous advances made in other cancer types. For example, an HER2 classifier obtained by MALDI IMS in breast cancer samples was valid to assess the HER2 status in gastric cancer with a sensitivity and specificity comparable to other techniques such as IHC or FISH [217]. A similar idea was recently explored in a broad pan-cancer analysis that included BC [218]. Although this HER2 index was formulated using a larger proportion of genomic data than protein information, it is of great interest because it also demonstrated a positive correlation between the affectations at these two levels.

Notably, there is strong evidence regarding the existence of a link between the subtype of BC and its estimated behavior and response to platinum-based therapies. Generally, MIBC basal tumors are more aggressive, whereas luminal tumors are more lenient. However, the behavior of the tumors and chemoresistance is unequal, because it was shown that luminal-p53 tumors were more resistant than basal-p53 tumors, which proved to be more sensitive to cisplatin [46]. In line with this, similar conclusions were obtained in another study that compared the response of luminal and basal BC to neoadjuvant cisplatin [54]. Here, patients with a luminal subtype had a better prognosis regardless of the administration of neoadjuvant chemotherapy, whereas the basal subtype had a worse outcome, and neoadjuvant cisplatin significantly improved the survival rate of those patients. Additionally, a recent publication by Mo, Q. et al. proposed a further subclassification of luminal and basal MIBC considering the presence of normal 9q21.3 chromosome fragment or their loss [219]. In general terms, the authors also found that patients with the basal subtype responded to neoadjuvant cisplatin; however, the subset that lost the 9q21.3 fragment did not respond to the addition of PD-L1 inhibitors to the therapeutic schema. Taken together, it seems reasonable to postulate that basal MIBC tumors have an accelerated cell cycle and more aggressive invading capabilities; however, because they also respond to chemotherapy, the presence of cisplatin temporarily blocks proliferation and cell motility, allowing other drugs the possibility to exert their effects. In summary, classifying BC in molecular subtypes promises the design of more effective, targeted first-line schemas based on platinum. However, the dynamics of the molecular mechanisms and cellular pathways that occur from initiating the therapy have not yet been elucidated, and proteomics could contribute to clarify this situation.

### 5.2. Response to Second-Line Therapies in Bladder Cancer

In clinics, it is just after the failure of first-line chemotherapy or suspected cisplatin-unfitting when alternative and second-line therapies are considered (Figure 10). These can be classified as immunotherapy or targeted chemotherapy. However, both are targeted therapies, because the former recurs to the use of specific antibodies, whereas targeted chemotherapy is focused on affecting the activity of a key protein by administering an inhibitor.

#### 5.2.1. Immunotherapy

Significant recent advances have been made in the immunotherapy of cancer, which contrast with the disappointing progress made in the past. This is not because these immunotherapies were ineffective, but because they did not fulfil the initial elevated expectative and the complexity of the dynamics of immuno-oncology. Since then, the landscape has significantly changed. Briefly, immunotherapy can be approached from the cellular or humoral levels. In cellular immunotherapy, immune cells are the central participants in the antitumoral response; thus, therapies are conceived to directly modulate their activity. This is the case of adoptive cell transfer (ACT) strategies such as CAR-T, and T-cell receptor (TCR)-engineered T-cells [220]. In contrast, humoral immunotherapy relies on administering antibodies to target a specific ligand; hence, the focus is more on promoting this biochemical interaction. Due to its simple conception, early immunotherapy can be ascribed to this group. Other schemas share features of both groups, such as the use of immune checkpoint inhibitors (ICIs), which are antibody-targeting ligands that participate in the inhibitory signaling pathways that cancerous cells profit for inducing immunoevasion.

Historically, in BC, immunotherapy has been successful. Administration of BCG in NMIBC is the standard of care. In MIBC, tumors exhibit upregulation of the programmed death-ligand 1/programmed cell death protein 1 (PD-L1/PD-1) signaling pathway. The FDA has approved nivolumab (anti-PD-1), pembrolizumab (anti-PD-1), atezolizumab (anti-PD-L1), durvalumab (anti-PD-L1), and avelumab (PD-L1), whereas the EMA has accepted nivolumab, pembrolizumab, and atezolizumab (Table 3). In addition, there are ongoing clinical trials exploring the utility of other ICIs such as ipilimumab, an anti-CTLA-4 antibody with 14 currently active studies related to BC.

In addition to these approved clinical antibodies, research of the PD-L1/PD-1 signaling pathway has allowed the development of novel antibodies such as sasanlimab, an anti-PD-L1 antibody, or tislelizumab, an anti-PD-1 antibody [230,231]. There are also ongoing clinical trials to explore the utility of other targets. This is the case for ipilimumab and tremelimumab; anti-CTLA-4 antibodies which are currently being tested in 14 active studies [232]; lirilumab, an anti-killer immunoglobulin-like receptor 2D (anti-KIR2D) that affects the activity of the immune NK-cells [233]; vofatamab, a promising anti-fibroblast growth factor receptor 3 (anti-FGFR3) which is a target commonly affected in BC [234]; catumaxomab, an anti-epithelial cell adhesion molecule (anti-Epcam) commonly expressed by ascites [235]; or panitumumab, an anti-epithelial growth factor receptor (anti-EGFR) [236]. In line with *ERBB* receptors, there have been interesting advances in the development of anti-HER2 antibodies. Earlier clinical trials employed cetuximab, which is usually prescribed for breast cancer. However, the results discouraged its application in BC either alone or in combination with chemotherapy [237,238]. At present, anti-HER2 antibody–drug conjugates (ADC) that exploit the advantage of targeted delivery of drugs such as trastuzumab, triplizumab, or vetixitumab and others are under investigation [239,240]. Moreover, research on enfortumab Vedotin-ejfv, an anti-Nectin 4 ADC recently approved for the treatment of BC, is also encouraging [241,242].

These therapeutic approaches acknowledge the importance of investigating the dynamics within the tumor microenvironment; therefore proteomics techniques that provide this information would be of great interest. A recent report explored changes to a B7-H4+ cell population that highlight the utility of mass cytometry [243]. B7-H4 belongs to the family of T-cell inhibitory regulators, together with the PD-L1/PD-1 and CTLA-4 signaling pathways. B7-H4 may be overexpressed in tumor cells and macrophages; thus, it is a potential target for the immunotherapy of BC as the study demonstrated.

Another novel study employed mass cytometry as a complementary technique to evaluate the cell population distribution within the tumor microenvironment after clustering patients as low- or high-immune using a panel of genes associated with tumor immune activity [244]. As expected, the immune cell population was quite heterogeneous. Of note, the authors also discovered a link between a higher extension of the immune suppression and the low-immune cluster, and this is an interesting finding that supports the contributions made by Sjödahl, G. et al. identifying the existence of pseudo-differentiation within a cluster as a consequence of immune infiltration [41,42].

Due to the key role played by the Treg-cell population in tumor immunoevasion, researchers have focused their interest on this area in recent years. This lineage is usually described as FOXP3+ T-cells, although CTLA-4 is also a marker found on them which is controlled by the expression of FOXP3. On the other hand, CTLA-4 is also an inhibitory immune checkpoint expressed by effector T-cells. Consequently, it has been hypothesized that tumor dosage with ipilimumab or tremelimumab, two clinically approved anti-CTLA-4 antibodies, would block the activity of these lineages, affecting the immunosuppressive activity of Treg-cells [245]. However, Sharma, A. et al. recently demonstrated, using IHC and mass cytometry, that the population of FOXP3+ T-cells remained largely unaffected in melanoma, prostate, and bladder cancer samples after treatment [246]. In conclusion, ICIs immunotherapies require further improvement.

Despite all these contributions, the popularity of mass cytometry in comprehending BC is still limited, and few studies exist in comparison with other more standardized techniques. Regarding to the contributions of proteomics to ACT strategies in BC, there are even fewer publications. However, this should not be interpreted as a boundary, because when we expanded our search to cancer research in general, we found probes based on contributions made by mass spectrometry-based proteomics [247,248,249,250,251,252,253].

#### 5.2.2. Targeted Chemotherapy

In 2019, the FDA granted the approval of erdafitinib in advanced BC, which became the first targeted chemotherapy available for treating this disease [254]. Erdafitinib is an inhibitor of FGFR3 activity, which is frequently affected in the luminal subtypes of BC. In contrast with immunotherapy, fewer drug candidates are currently in advanced clinical research stages beyond phase I, with rogaratinib, olaparib, vistusertib, and cabozantinib being promising candidates to be adopted in the clinical practice [255,256,257,258]. The former is a broader inhibitor of the FGFRs, whereas olaparib blocks the activity of Poly-(ADP-ribose) Polymerase (PARP) that assists in repairing damaged DNA; vistusertib affects the mTOR cell proliferating pathway (Figure 3), and the latter is an angiogenic inhibitor of the vascular endothelial growth factor receptor-2 (VEGFR2) pathway. This situation may be explained because they are more prone to cause more side effects and a shorter duration than ICIs, although the response rate is equally promising, at least these were the conclusion with erdafitinib [254]. Likewise, a phase III clinical trial with lapatinib, an EGFR/HER2 tyrosine kinase inhibitor, was discontinued due to significant adverse effects [259]. Moreover, other EGFR inhibitors such as gefitinib were abandoned due to a lack of significant improvement in patient outcomes [260].

The challenging advances in the applicability of targeted chemotherapy for managing BC are mimicked by the few contributions we found from proteomics. This was surprising, because mass spectrometry is highly appropriate for elucidating the changes arising in the phosphoproteome as a consequence of the activity exerted by tyrosine kinase inhibitors. This was the case when we explored the activity of the aforementioned inhibitors in other cancer types, such as cholangiocarcinoma, breast cancer, hepatocellular cancer, or gastric cancer [261,262,263,264]. However, the conclusions and findings may not be extrapolated to BC; thus, further research is strongly needed.

## 6. Conclusions and Perspectives

This review recapitulates the current knowledge regarding the molecular pathophysiology of BC and the capacity of clustering BC in subtypes. These advances support a better understanding of the molecular mechanisms governing BC and will potentially shift the view from a heterogeneous disease towards more specific and diverse BC subtypes. In addition, this molecular classification of BC should enable the development of targeted therapies with properly designed clinical trials. In this context, proteomics offers additional information on the behavior of the tumor, especially when we need to envisage the role of the immune system or tumor advancement through the different tissue layers. The existence of proteomic subclusters within a genomic subtype should not be omitted to acquire a better understanding of cell interactions. Nevertheless, antibody-based techniques, such as ELISA or immunohistochemistry, currently dominate the arsenal of proteomic techniques because they are usually preferred in clinical routines. In contrast, mass spectrometry-based techniques are considered more investigational and their implementation is several steps behind in comparison with other massive analytical techniques, such as NGS or gene microarrays. An effort should be made to proffer techniques such as LMD-LC-MS or MALDI-MSI as a grounding for more advanced techniques that have appeared in recent years, such as single-cell proteomics.

This review has highlighted the enormous potential that proteomics and particularly mass spectrometry-based techniques, in general, play in BC. On the one hand, classic approaches have enabled the identification and quantification of a vast number of proteins, allowing the focus on a region of interest when adjoined with tools such as LMD. In addition, SRM-MS and MRM-MS offer great sensitivity and accuracy for quantifying a selection of proteins of interest; hence, they are an alternative to antibody-based methods with the added advantage of being high-throughput methods that allow the simultaneous quantification of several proteins. On the other hand, the investigation of proteins—and the functional networks in which they participate—enables the design of panels to discover how tumoral cells function and the related molecular dynamics that support this. The study of the ECM, inflammation processes, EMT, angiogenesis and vasculogenesis, cell metabolism, cell differentiation, or cell adhesion are key processes that can be linked with relevant clinical information such as the outcomes or the responses of BC to selected therapies. The most all-embracing strategy that can manage this information is the development of proteomics clusters. In addition, the development of novel mass spectrometry-based proteomics techniques, focused on maintaining the spatial information, provides an in-depth, promising perspective on the topology of the tumor by revealing the localization and levels of a broad range of proteins. The possibilities that arise from combining these three realms are both challenging and stimulating. Finally, it is intriguing how proteomics has been, in the last five years, a step behind in the study of patient responses to therapies. This situation contrasts with the dynamic contribution found in other cancer types, and we aim to seriously consider proteomics as a valuable option.

## Figures and Tables

**Table 1:** TNM (tumor–node–metastasis) classification of bladder cancer.

**T—Primary tumor**		
Tx	Primary tumor cannot be assessed	
T0	No evidence of primary tumor	
Ta	Non-invasive papillary carcinoma	
Tis	Carcinoma in situ: “flat tumor”	
T1	Tumor invades subepithelial connective tissue	
T2	Tumor invades muscle:	
	T2a	Tumor invades superficial muscle (inner half)
	T2b	Tumor invades deep muscle (outer half)
T3	Tumor invades perivesical tissue:	
	T3a	Microscopically
	T3b	Macroscopically (extravesical mass)
T4	Tumor invades surrounding organs:	
	T4a	Tumor invades prostate stroma, seminal vesicles, uterus, or vagina
	T4b	Tumor invades pelvic wall or abdominal wall
**N—Regional lymph nodes**		
Nx	Regional lymph nodes cannot be assessed	
N0	No regional lymph node metastasis	
N1	Metastasis in a single lymph node in the pelvis	
N2	Metastasis in multiple regional lymph nodes in the pelvis	
N3	Metastasis in a common iliac lymph node(s)	
**M—Distant metastasis**		
M0	No distant metastasis	
M1	Distant metastasis:	
	M1a	Non-regional lymph nodes
	M1b	Other distant metastasis

**Table 2:** Comparison between the third (2004) and the recent fourth (2016) WHO classifications of tumors of the urothelial tract. *, still used informally; -, not present in the corresponding version.

Urothelial-Type Tumors				Non-Urothelial-Type Tumors	
Non-Invasive		Invasive			
WHO 2004	WHO 2016	WHO 2004	WHO 2016	WHO 2004	WHO 2016
Urothelial carcinoma in situ		With squamous differentiation	*	Squamous cell neoplasm	
Papillary urothelial carcinoma, low grade		With glandular differentiation	*	Glandular neoplasm	
Papillary urothelial carcinoma, high grade		With small cell differentiation	*	-	Urachal carcinoma
Papillary urothelial neoplasm of low malignant potential		With trophoblastic differentiation	*	-	Tumors of Müllerian type
Urothelial papilloma		Nested		Neuroendocrine tumors	
Inverted urothelial papilloma		Microcystic		Melanocytic tumors	
-	Urothelial proliferation of uncertain malignant potential (hyperplasia)	Micropapillary		Mesenchymal tumors	
-	Urothelial dysplasia	Lymphoepithelioma-like		Urothelial tract hematopoietic and lymphoid tumors	
		Lymphoma-like	-	Miscellaneous	
		Plasmacytoid	Plasmacytoid/signet ring cell/diffuse		
		Sarcomatoid			
		Giant cell			
		Undifferentiated	Poorly differentiated		
		-	Lipid rich		
		-	Clear cell		

**Table 3:** Clinical trials using approved ICI immunotherapy in the treatment of bladder cancer.

Patients/Phase	Schema	Setting	Study Conclusions	Year, [Ref.]
386/II	Nivolumab 3 mg/kg IV	mMBIC	OS was 7.0 months, and effectiveness was irrespective of PD-L1 expression levels	2017, [221]
119/II	Carboplatin vs. Atezolizumab 1200 mg IV	mMIBC	OS was 15.0 months with atezolizumab, and 12.1 months with any platinum-based chemotherapy, and 8.7 months with carboplatin/gemcitabine. First-line atezolizumab for cisplatin-unfit mMIBC provided an OS benefit over platinum-based treatments	2019, [222]
310/II	Atezolizumab 1200 mg IV	mMIBC	Increased levels of PD-L1 were associated with better responses. All TCGA 2014 subtypes responded to the therapy, although it was significantly higher in the luminal cluster II	2016, [223]
931/III	Vinflunine, paclitaxel, or docetaxel vs. Atezolizumab 1200 mg IV	mMIBC	Updated OS demonstrated long-term stable remission. After 24 months, the OS was 23% to atezolizumab, and 13% to the alternative chemotherapy	2021, [224]
39/II	GC + Pembrolizumab 200 mg IV	MIBC (T2-4a, N0-1, M0)	With neoadjuvant GC + pembrolizumab, 56% of patients (95% CI, 40 to 72) achieved <pT2N0, and 36% (95% CI, 21 to 53) achieved pT0N0	2021, [225]
542/III	Vinflunine, paclitaxel, or docetaxel vs. pembrolizumab 200 mg IV	mMIBC	After 24 months of follow-up, long-term outcomes were better with pembrolizumab over chemotherapy	2019, [226]
374/II	Pembrolizumab 200 mg IV	mMIBC	8.9%, and 19.7% of patients achieved complete and partial response, respectively, with a median of 30.1 months (95% CI). Patients with unaffected lymph nodes had better outcome rates	2020, [227]
44/Ib	Avelumab 10 mg/kg IV	mMIBC	Avelumab was safe, and it was associated with a large median duration of response, and a prolonged survival rate	2017, [228]
249/I	Avelumab IV 10 mg/kg IV	mMIBC	Avelumab as neoadjuvant in platinum-treated patients was safe, and demonstrated the best overall complete or partial response	2018, [229]

IV: intravenous; mMIBC: advanced metastatic muscle invasive bladder cancer; OS: overall survival rate; TCGA: The Cancer Genome Atlas Program; CI: confidence interval; GC: gemcitabine/cisplatin.
